# “De novo replication repair deficient glioblastoma, IDH-wildtype” is a distinct glioblastoma subtype in adults that may benefit from immune checkpoint blockade

**DOI:** 10.1007/s00401-023-02654-1

**Published:** 2023-12-11

**Authors:** Sara Hadad, Rohit Gupta, Nancy Ann Oberheim Bush, Jennie W. Taylor, Javier E. Villanueva-Meyer, Jacob S. Young, Jasper Wu, Ajay Ravindranathan, Yalan Zhang, Gayathri Warrier, Lucie McCoy, Anny Shai, Melike Pekmezci, Arie Perry, Andrew W. Bollen, Joanna J. Phillips, Steve E. Braunstein, David R. Raleigh, Philip Theodosopoulos, Manish K. Aghi, Edward F. Chang, Shawn L. Hervey-Jumper, Joseph F. Costello, John de Groot, Nicholas A. Butowski, Jennifer L. Clarke, Susan M. Chang, Mitchel S. Berger, Annette M. Molinaro, David A. Solomon

**Affiliations:** 1 Department of Neurological Surgery, University of California, San Francisco, San Francisco, CA USA; 2 Department of Pathology, University of California, San Francisco, San Francisco, CA USA; 3 Department of Neurology, University of California, San Francisco, San Francisco, CA USA; 4 Department of Radiology and Biomedical Imaging, University of California, San Francisco, San Francisco, CA USA; 5 Department of Radiation Oncology, University of California, San Francisco, CA USA

**Keywords:** Giant cell glioblastoma, Hypermutation, Ultrahypermutation, Mismatch repair deficiency, *POLE*, Lynch syndrome, Immune checkpoint blockade, Immunotherapy, Molecular neuropathology, Molecular neuro-oncology

## Abstract

Glioblastoma is a clinically and molecularly heterogeneous disease, and new predictive biomarkers are needed to identify those patients most likely to respond to specific treatments. Through prospective genomic profiling of 459 consecutive primary treatment-naïve IDH-wildtype glioblastomas in adults, we identified a unique subgroup (2%, 9/459) defined by somatic hypermutation and DNA replication repair deficiency due to biallelic inactivation of a canonical mismatch repair gene. The deleterious mutations in mismatch repair genes were often present in the germline in the heterozygous state with somatic inactivation of the remaining allele, consistent with glioblastomas arising due to underlying Lynch syndrome. A subset of tumors had accompanying proofreading domain mutations in the DNA polymerase *POLE* and resultant “ultrahypermutation”. The median age at diagnosis was 50 years (range 27–78), compared with 63 years for the other 450 patients with conventional glioblastoma (*p* < 0.01). All tumors had histologic features of the giant cell variant of glioblastoma. They lacked *EGFR* amplification, lacked combined trisomy of chromosome 7 plus monosomy of chromosome 10, and only rarely had *TERT* promoter mutation or *CDKN2A* homozygous deletion, which are hallmarks of conventional IDH-wildtype glioblastoma. Instead, they harbored frequent inactivating mutations in *TP53*, *NF1*, *PTEN*, *ATRX*, and *SETD2* and recurrent activating mutations in *PDGFRA*. DNA methylation profiling revealed they did not align with known reference adult glioblastoma methylation classes, but instead had unique globally hypomethylated epigenomes and mostly classified as “Diffuse pediatric-type high grade glioma, RTK1 subtype, subclass A”. Five patients were treated with immune checkpoint blockade, four of whom survived greater than 3 years. The median overall survival was 36.8 months, compared to 15.5 months for the other 450 patients (*p* < 0.001). We conclude that “De novo replication repair deficient glioblastoma, IDH-wildtype” represents a biologically distinct subtype in the adult population that may benefit from prospective identification and treatment with immune checkpoint blockade.

**Supplementary Information:**

The online version contains supplementary material available at 10.1007/s00401-023-02654-1.

## Introduction

“Glioblastoma, IDH-wildtype” (hereafter glioblastoma) remains a clinically, histologically, genetically, and epigenetically heterogeneous disease despite significant definitional updates in the 2021 World Health Organization (WHO) Classification of Central Nervous System Tumors aimed at homogenizing it into a more biologically unified tumor type [32]. Specifically, glioblastoma is now considered an adult-type diffuse glioma that occurs in adults over 25 years of age in the vast majority of cases. However, a specific age cut-off between “diffuse pediatric-type high-grade glioma” and adult-type glioblastoma has not been established, and a variable patient-specific gray zone may exist for high-grade gliomas occurring during the second to fourth decades of life. Glioblastoma is now also defined explicitly as an IDH-wildtype and histone H3-wildtype diffuse astrocytic glioma, eliminating all IDH-mutant astrocytomas regardless of their WHO grade and histologic features morphologically resembling glioblastoma, as well as eliminating H3 K27-altered diffuse midline gliomas and H3 G34-mutant diffuse hemispheric gliomas. Furthermore, glioblastoma is now exclusively considered to arise as a primary de novo neoplasm, and the concept of “secondary glioblastoma” arising from malignant transformation of diverse lower-grade glioma entities has been eliminated. Lastly, molecular profiling has revealed that many tumors originating in the thalamus and infratentorial midline structures (e.g., cerebellum, brainstem, and spinal cord) previously diagnosed histologically as glioblastoma represent other WHO Classification recognized tumor entities such as high-grade astrocytoma with piloid features (HGAP) or H3 K27-altered diffuse midline gliomas [40, 42]. While these updates in the classification and definition of glioblastoma have indeed resulted in a more biologically homogenous group of tumors, it remains a clinically and molecularly diverse disease, with some patients experiencing rapid disease progression and others experiencing long-term survival beyond 5 years despite identical treatment with maximal safe resection, external beam radiation, and adjuvant chemotherapy with the alkylating agent temozolomide [38]. As such, new predictive biomarkers are needed to identify those patients most likely to respond to specific treatment regimens being developed and tested in ongoing clinical trials.

Immune checkpoint blockade has emerged over the past two decades as a therapeutic strategy that activates immune cells to better recognize cancer cells as being antigenically foreign, thus promoting their clearance by the immune system [37]. The programmed cell death protein 1 (PD-1, also called CD279) is a transmembrane receptor on immune cells that is activated by its ligand PD-L1 (also called CD274) on the surface of antigen-presenting cells that functions to inhibit immune cell activity upon receptor binding. Some cancers express high levels of PD-L1 as a mechanism to suppress recognition and clearance by the immune system. Humanized monoclonal antibodies that bind and inhibit the PD-1 receptor or the PD-L1 ligand have demonstrated significant efficacy in treating specific cancer types. Several of these agents, including pembrolizumab, nivolumab, and others, are now approved by the United States Food and Drug Administration (FDA) and the European Medicines Agency (EMA) for treating patients with advanced or refractory cancer. An important biomarker of patient response to immune checkpoint blockade that has emerged is high somatic tumor mutation burden (TMB), which is a quantitative measure of the number of somatic (tumor-specific) nonsynonymous mutations across the coding exome of a cancer. Tumors with a high somatic TMB have an increased number of neoantigens present on their cell surface as a result, which can facilitate tumor cells being recognized as antigenically foreign and cleared by the immune system. Multiple prospective clinical trials have demonstrated that cancers with high TMB are those which most often have successful immune clearance and prolonged patient survival during treatment with immune checkpoint blockade agents [2, 28, 29]. Unfortunately, most patients with glioblastoma and other glioma subtypes have low numbers of somatic mutations (low TMB values) and do not demonstrate sustained responses to immune checkpoint blockade [30, 36, 39]. However, recent studies have identified that there is a small subset of gliomas with high TMB, and individual patients with “hypermutated” gliomas have been reported to show exceptional responses to immune checkpoint blockade [9, 20, 25, 46].

Two major sources of high somatic TMB in cancers are mismatch repair deficiency and DNA polymerase proofreading deficiency, which are collectively termed replication repair deficiency [1, 12]. Disruption of the mismatch repair protein complex, consisting of MSH2, MSH6, MLH1, and PMS2 subunits, is one the major causes of genetic instability that underlies a significant fraction of specific cancer types, particularly colorectal and endometrial carcinomas. Deleterious mutations that inactivate one of these canonical mismatch repair (MMR) genes result in mismatch repair deficiency that causes “hypermutation” of the cancer genome with a characteristic mutational signature that is predominated by C > T:G > A transitions, C > A:G > T transversions, and small (less than 3 base pair) insertions and deletions at mononucleotide and polynucleotide repeat sequences [1, 12]. The deleterious mutations in MMR genes can either be somatic (tumor-acquired) in origin or transmitted in the germline and present constitutionally in all of the cells in the body. Such constitutional mutations present on one of two alleles in the heterozygous state are causative of Lynch syndrome (also termed hereditary nonpolyposis colorectal cancer [HNPCC]), an autosomal dominant tumor predisposition syndrome with increased incidence of colorectal, endometrial, and upper urothelial tract carcinomas, sebaceous neoplasms, and occasionally malignant gliomas [33]. The malignant gliomas that arise in the setting of Lynch syndrome have been reported to include both IDH-wildtype glioblastomas and IDH-mutant astrocytomas, although the precise nature of these syndromic gliomas has yet to be fully defined [8, 41, 45, 46]. Homozygous or compound heterozygous germline mutations causing biallelic constitutional inactivation of an MMR gene result in constitutional mismatch repair deficiency (CMMRD), an autosomal recessive tumor predisposition syndrome characterized by café-au-lait macules and the development of diffuse pediatric-type high grade gliomas, T-cell lymphomas, and colorectal cancer during childhood [5]. In addition to the biallelic germline mutations in an MMR gene causing mismatch repair deficiency, these childhood tumors arising in the setting of CMMRD often acquire mutations in the proofreading domain of DNA polymerase epsilon (*POLE*) or delta (*POLD1*). These proofreading domain mutations in *POLE* and *POLD1* are known to synergize with mismatch repair deficiency to cause a rapid burst of a massive number of substitution mutations causing “ultrahypermutation” of the cancer genome with a characteristic mutational signature that is predominated by C > A:G > T transversions specifically at cytosines with flanking thymine bases (TCT nucleotide sequence) [12, 43]. The ultrahypermutated diffuse pediatric-type high grade gliomas arising in the setting of CMMRD have distinct hypomethylated epigenomes, frequent mutational activation of the MAP kinase signaling pathway, and often demonstrate remarkable responses to immune checkpoint blockade that have resulted in tumor clearance and long-term survival for affected children [9, 11, 17–19]. Rare cases of IDH-wildtype glioblastoma occurring in adult patients with similar proofreading domain missense mutations in *POLE*, ultrahypermutation, and response to immune checkpoint blockade have also been reported, although their precise frequency remains uncertain [6, 7, 13, 20, 24].

Here, we have performed prospective genomic profiling on a large cohort of primary treatment-naïve IDH-wildtype glioblastomas in adults. This enabled our identification and further in-depth study of a unique subgroup defined by somatic hypermutation and DNA replication repair deficiency (RRD) due to biallelic inactivation of a canonical mismatch repair gene, with a subset having accompanying *POLE* proofreading domain mutation and ultrahypermutation. Our results provide evidence that “De novo replication repair deficient glioblastoma, IDH-wildtype” should be regarded as a new distinct subtype of IDH-wildtype glioblastoma in the adult population based on its unique mechanism of oncogenesis, underlying genetic drivers, epigenomic profile, and cellular composition. The findings in our cohort, together with prior case reports, suggest that prospective identification and treatment with immune checkpoint blockade may improve survival for affected patients.

## Methods

### Patient cohort and tumor samples

The study cohort consisted of 459 consecutive adult patients over 25 years of age who underwent surgical biopsy or resection of an initial primary treatment-naïve IDH-wildtype glioblastoma at the University of California, San Francisco between 2017 and 2022. All patients had tumors pathologically confirmed as glioblastoma, IDH-wildtype according to the 2021 WHO Classification of Central Nervous System Tumors based on the combination of histopathologic features and targeted next-generation DNA sequencing including assessment of gene mutations, fusions and other structural variants, and chromosomal copy number profiles. All tumors were diffuse astrocytic gliomas located supratentorially in the cerebral hemispheres with histologic features of glioblastoma (i.e., containing necrosis and/or microvascular proliferation) and were confirmed to be IDH-wildtype (i.e., lacking *IDH1* p.R132 and *IDH2* p.R172 mutation), histone H3-wildtype (i.e., lacking p.K27 or p.G34 mutation in *H3F3A* [now *H3-3A*], *H3F3B* [now *H3-3B*], *HIST1H3B* [now *H3C2*], and *HIST1H3C* [now *H3C3*]), and an absence of an overall molecular profile indicative of another tumor type (i.e. lacking *KIAA1549*::*BRAF* or *ZFTA*::*RELA* fusion). Patients with a history of prior therapeutic radiation for childhood malignancy (i.e., radiation-induced gliomas) or any prior lower-grade glioma were excluded, as were patients with infratentorial gliomas centered in the cerebellum, brainstem, and spinal cord, as these are likely to represent other biologically distinct tumor types. This study was approved by the institutional review board of the University of California, San Francisco. As part of routine clinical practice at UCSF, all patients in this study signed a written waiver of informed consent to contribute de-identified data to research projects.

### Targeted next-generation DNA sequencing

Prospective genomic evaluation was performed on a clinical basis in a CLIA-certified laboratory for all tumors using the UCSF500 NGS Panel as previously described [27, 51], which typically provides greater than 500 × sequencing coverage over the *IDH1* p.R132 and *IDH2* p.R172 mutational hotspots, as well as providing comprehensive assessment of cytogenetic alterations (e.g., chromosomes 1p and 19q status, chromosomes 7 and 10 status) and genetic alterations (e.g.,* EGFR*, *PDGFRA*, *MET*, *FGFR3*, *NF1*, *BRAF*, *PIK3CA*, *PIK3R1*, *PTEN*, *CDKN2A*, *CDK4*, *CDK6*, *RB1*, *TP53*, *MDM2*, *MDM4*, *H3F3A*, *HIST1H3B*, *CIC*, *FUBP1*, *TERT* [including promoter region], *ATRX*) critical for glioma diagnostic assessment. Tumor tissue was selectively scraped from unstained slides or punched from formalin-fixed, paraffin-embedded blocks using biopsy punches to enrich for high tumor content (> 25% tumor fraction). Genomic DNA was extracted from this macrodissected formalin-fixed, paraffin-embedded tumor tissue using the QIAamp DNA FFPE Tissue Kit (Qiagen). For a subset of patients, as indicated, a constitutional DNA sample was extracted from a buccal swab or peripheral blood specimen and simultaneously sequenced to enable accurate discrimination of germline versus somatic origin of variants. Capture-based next-generation DNA sequencing was performed using an assay that targets all coding exons of 529 cancer-related genes, select introns and upstream regulatory regions of 73 genes to enable detection of structural variants including gene fusions, and DNA segments at regular intervals along each chromosome to enable genome-wide copy number and zygosity analysis, with a total sequencing footprint of 2.8 Mb. Multiplex library preparation was performed using the KAPA Hyper Prep Kit (Roche) according to the manufacturer’s specifications. Hybrid capture of pooled libraries was performed using a custom oligonucleotide library (Integrated DNA Technologies). Captured libraries were sequenced as 150 bp paired-end reads on an Illumina NovaSeq 6000 instrument. Sequence reads were mapped to the reference human genome build GRCh37 (hg19) using the Burrows-Wheeler aligner (BWA). Recalibration and deduplication of reads was performed using the Genome Analysis Toolkit (GATK). Coverage and sequencing statistics were determined using Picard CalculateHsMetrics and Picard CollectInsertSizeMetrics. Single nucleotide variant and short insertion/deletion mutation calling was performed with Mutect2, FreeBayes, Unified Genotyper, and Pindel. Larger insertion/deletion and structural alteration calling was performed with Pindel and Delly. Variant annotation was performed with Annovar. Single nucleotide variants, insertions/deletions, and structural variants were visualized and verified using Integrative Genome Viewer. Genome-wide copy number and zygosity analysis was performed by CNVkit and visualized using NxClinical (BioDiscovery). Microsatellite instability determination was performed with MSIsensor2 analysis of mononucleotide and dinucleotide repeats [35]. Glioblastomas were deemed positive for microsatellite instability when ≥ 10% of the 86 microsatellites assessed by the UCSF500 NGS Panel were unstable. Somatic tumor mutation burden (TMB) was determined by calculating the number of somatic mutations in the coding regions of genes in the UCSF500 NGS Panel, counting both single nucleotide variants and short indels, divided by the total coding footprint of the assay (1.5 Mb). For patients with paired tumor-normal sequencing performed, TMB was calculated using only the confirmed somatic mutations. For patients with tumor-only sequencing performed, TMB was calculated by removing known germline variants present at ≥ 0.001% frequency in human population datasets (ExAC, gnomAD, and NHLBI-ESP6515). Hypermutation was defined as those tumors with TMB values of ≥ 15 somatic mutations per Mb based on this assay. Each of the confirmed somatic variants from paired tumor-normal sequencing or filtered variants from tumor-only sequencing analysis were reviewed for predicted pathogenicity using known cancer genomics data in the COSMIC (http://cancer.sanger.ac.uk/cosmic) and cBioPortal (http://www.cbioportal.org/) databases, location within encoded protein, and predicted functional effect based on mutation type (e.g., missense, nonsense, frameshift, splice site).

### Genome-wide DNA methylation profiling

Genomic DNA from 105 adult IDH-wildtype glioblastomas (98 conventional glioblastoma, IDH-wildtype and 7 “de novo replication repair deficient glioblastoma, IDH-wildtype”) was bisulfite converted using the EZ DNA Methylation kit following the manufacturer’s recommended protocol (Zymo Research). Bisulfite converted DNA was amplified, fragmented, and hybridized to Infinium EPIC 850k Human DNA Methylation BeadChips following the manufacturer’s recommended protocol (Illumina). Methylation data were preprocessed using the minfi package (v.1.38.0) in R Bioconductor (v.3.5.3) [3]. The detection *p*-value for each sample was computed, and CpG sites with detection *p*-values above 0.05 were discarded from the analysis. Functional normalization with NOOB background correction and dye-bias normalization was performed with the preprocessFunnorm function [21, 47]. Probe filtering was performed after normalization. Specifically, probes located on sex chromosomes, containing nucleotide polymorphisms (dbSNP132 Common) within five base pairs of and including the targeted CpG site, or mapping to multiple sites on hg19 (allowing for one mismatch), as well as cross-reactive probes were removed from analysis. The dmpFinder function from the minfi package (v.1.38.0) was applied on the β-value matrix to identify the 5000 most differentially methylated CpG sites among the cohort of 105 IDH-wildtype glioblastomas. Pearson distance matrix with complete linkage was used to plot a heatmap with visualization performed using the R package ComplexHeatmap (v.2.0.0) [22]. A violin plot was generated showing the mean β-value for each of the approximately 850,000 CpG sites across the de novo RRD and conventional glioblastoma groups using ggplot2 (v.3.4.2). Random forest classification of DNA methylation profiles was performed to compare each tumor’s epigenetic signature against established reference methylation classes of CNS tumors as previously described using the DKFZ MolecularNeuropathology.org online classifier (v.12.7) [14, 15]. The DNA methylation profiles of 7 de novo RRD glioblastomas were also assessed together with 1143 reference samples spanning 25 CNS tumor methylation groups and 3 control tissue methylation groups previously generated at DKFZ (sample manifest in Supplementary Table S7) [14]. Since a subset of the reference cohort contained methylation data generated using the Infinium Human Methylation 450k BeadChips, the approximately 450,000 overlapping CpG sites between the EPIC 850k and 450k BeadChips were used in the analysis. A beta-value matrix with approximately 379,400 CpG probes was used for analysis. Row-wise standard deviation was calculated for each probe across all samples, and the 30,000 most differentially methylated probes were selected. Dimensionality reduction using t-distributed stochastic neighbor embedding (tSNE) was performed by Rtsne (v.0.15) using the following analysis parameters: dims = 2, max_iter = 5000, theta = 0, perplexity = 30. The tSNE plot was visualized with ggplot2 (v.3.4.2).

### Chromosomal copy number analysis

In addition to the chromosomal copy number data obtained from the targeted next-generation DNA sequencing platform, chromosomal copy number profiles were derived from the Infinium EPIC DNA methylation array data using the conumee package (v.1.26.0) [23]. Summary copy number profiles for the de novo RRD glioblastoma and conventional glioblastoma groups were generated using a custom script modified from the version previously used for CNS tumor summary CNV plots [15]. Chromosomal gains and losses were counted when the intensity ratio of a chromosomal segment (binned by 300 consecutive CpG sites) deviated from the baseline by more than ± 0.1.

### Immune cell deconvolution from DNA methylation array data

We used methylCIBERSORT as previously described [16, 34] to estimate neoplastic cell and various tumor microenvironment cell fractions in each sample using a custom CNS tumor signature matrix previously generated at the National Cancer Institute [44]. The deconvoluted cell fractions were visualized with ggplot (v.3.4.2).

### Immune checkpoint blockade treatment

An FDA-approved humanized monoclonal antibody (pembrolizumab or nivolumab) targeting the PD-1 receptor was used off-label to treat five of the patients in this de novo RRD glioblastoma study cohort, with a sixth patient initiating treatment at the time of manuscript drafting. Recommended intravenous dosing was followed (generally 200 mg every 3 weeks for pembrolizumab and 480 mg every 4 weeks for nivolumab), and interval assessment for known toxicities and treatment response was performed.

### Statistical analysis

Patient demographics and tumor characteristics were summarized with descriptive statistics. Student’s *t*-tests and *χ*^2^ tests were used to compare continuous and categorical variables between patient cohorts, respectively, with a *p*-value less than 0.05 considered significant. Poisson distribution *z*-test was used to compare genetic alteration frequency between the two tumor groups, with a *p*-value less than 0.05 considered significant. The genetic alteration frequency between the two tumor groups was also compared using *χ*^2^ tests that produced identical significance results to the *z*-tests. Differences in cellular composition between the two tumor groups were compared using Mann–Whitney *U* test, with a *p*-value less than 0.05 considered significant. Overall survival was defined as the time from the initial diagnostic surgical procedure until death or censoring at the last clinical follow-up visit. Kaplan–Meier plots were used to visualize survival stratified by the two tumor groups, and differences in survival were determined by log-rank test. Median overall survival times and 95% confidence interval were estimated using the Kaplan–Meier method. All analyses were conducted using the statistical software R version 4.2.3 (http://www.r-project.org/).

## Results

### Identification of a novel glioblastoma, IDH-wildtype subtype in adults

Our prospective genomic profiling of 459 consecutive primary treatment-naïve IDH-wildtype glioblastomas within the cerebral hemispheres of adults identified a distinct subgroup defined by the combination of somatic hypermutation (TMB of ≥ 15 somatic mutations per Mb) and biallelic inactivation of a canonical mismatch repair gene (*MSH2*, *MSH6*, *MLH1*, or *PMS2*) with loss of the affected mismatch repair protein by immunohistochemistry (Table 1, Supplementary Tables S1 and S2). This novel tumor subgroup that we provisionally designated “De novo replication repair deficient glioblastoma, IDH-wildtype” accounted for 2% of the patient cohort (9/459 cases).

**Table 1:** Clinicopathologic features of "De novo replication repair deficient glioblastoma, IDH-wildtype" in comparison to conventional glioblastoma, IDH-wildtype

	Total GBM, IDH-wildtype cohort (*n* = 459)	Conventional GBM, IDH-wildtype (*n* = 450)	De novo RRD GBM, IDH-wildtype (*n* = 9)	*p* value
Sex				0.13
Female	193 (42%)	187 (42%)	6 (67%)	
Male	266 (58%)	263 (58%)	3 (33%)	
Age				< 0.01
Mean	62 yrs	63 yrs	50 yrs	
Median	63 yrs	63 yrs	50 yrs	
Q1, Q3	55, 71	55, 71	40, 57	
Range	26–94	26–94	27–78	
Tumor location				0.78
Cerebral hemispheres	455 (99%)	446 (99%)	9 (100%)	
Basal ganglia/Thalamus	4 (1%)	4 (1%)	0 (0%)	
Brainstem	0 (0%)	0 (0%)	0 (0%)	
Cerebellum	0 (0%)	0 (0%)	0 (0%)	
Spinal cord	0 (0%)	0 (0%)	0 (0%)	
*IDH1/2* status				N/A
Mutant	0 (0%)	0 (0%)	0 (0%)	
Wildtype	459 (100%)	450 (100%)	9 (100%)	
Histone H3 K27 and G34 status				N/A
Mutant	0 (0%)	0 (0%)	0 (0%)	
Wildtype	459 (100%)	450 (100%)	9 (100%)	
Tumor mutation burden (somatic muts/Mb)				< 0.01
Mean	5	2	119	
Median	1	1	36	
Q1, Q3	1, 5	1, 5	24, 141	
Range	0–550	0–14	15–550	
*MGMT* promoter methylation status				0.17
Methylated	267 (67%)	264 (67%)	3 (43%)	
Unmethylated	132 (33%)	128 (33%)	4 (57%)	
Missing/not tested	60	58	2	

Four of the nine patients were genetically confirmed to have an inactivating/pathogenic germline mutation in one of the mismatch repair genes (three *MSH2*, one *MSH6*) in the heterozygous state with somatic tumor-acquired inactivation of the remaining allele due to either loss of heterozygosity or a second mutation present in trans (Supplementary Table S3). This is consistent with glioblastomas arising due to underlying Lynch syndrome in these patients, some of whom had a known family history of colorectal cancer and Lynch syndrome while others were newly diagnosed. Three of the nine patients were genetically confirmed to have exclusively somatic inactivation of the mismatch repair gene in the tumor with absence of the underlying mutation(s) in a matched constitutional DNA sample, thus indicating this de novo RRD glioblastoma subtype can also occur sporadically in the absence of underlying Lynch syndrome. For the remaining two patients, genomic testing was performed on tumor tissue only without analysis of a constitutional DNA sample that precluded definitive assessment of somatic versus germline origin of the mismatch repair gene mutation. However, underlying Lynch syndrome was clinically suspected in at least one of these two patients (#7) with a parent who had died of glioblastoma. None of the adult patients with IDH-wildtype glioblastomas in this large cohort were found to have biallelic germline mutation in a mismatch repair gene indicative of constitutional mismatch repair deficiency (CMMRD) syndrome due to homozygous or compound heterozygous germline mutations in one of the mismatch repair genes, which is associated with the development of an IDH- and histone H3-wildtype diffuse pediatric-type high-grade glioma subtype arising during childhood in most instances [5, 43].

In a subset of the de novo RRD glioblastomas (3/9, 33%), the mismatch repair deficiency was accompanied by a somatic *POLE* missense mutation in the proofreading domain of the encoded DNA polymerase epsilon: p.A456P, p.S461P, and p.V411L (annotated per RefSeq transcript NM_006231). These were all pathogenic ‘hotspot’ mutations known to disrupt the replication repair activity of DNA polymerase epsilon and corresponded with “ultrahypermutation” in these three tumors with TMB of approximately 150 to 500 somatic mutations per Mb. While the mutational signature of the other six de novo RRD glioblastomas was predominantly composed of C > T:G > A transitions and small indel mutations consistent with mutagenesis caused by underlying mismatch repair deficiency, the mutational signature in the three ultrahypermutated de novo RRD glioblastomas with accompanying *POLE* mutations featured a major component of C > A:G > T transversions occurring at cytosines with flanking thymine bases consistent with mutagenesis caused by underlying DNA polymerase proofreading deficiency (Supplementary Table S3) [1, 12, 43]. No glioblastomas in this large prospective cohort had *POLE* proofreading domain mutations occurring in isolation without accompanying mismatch repair deficiency.

Microsatellite stability analysis was performed by MSIsensor2 assessing 86 microsatellites per tumor as part of the next-generation DNA sequencing assay. Each of the nine de novo RRD glioblastomas demonstrated a low to moderate level of microsatellite instability, with instability at 10–30% of the evaluated microsatellites. This was in contrast to the 450 conventional IDH-wildtype glioblastomas that were universally microsatellite stable and had instability at less than 5% of the evaluated microsatellites. Notably, the de novo RRD glioblastomas had lower levels of microsatellite instability than mismatch repair deficient colorectal and endometrial carcinomas by the same assay that usually demonstrate instability at 30–60% of the evaluated microsatellites (data not shown). Prior studies have documented that CMMRD-associated diffuse pediatric-type high-grade gliomas often display only low levels of microsatellite instability similar to what we observed in our cohort of de novo RRD glioblastomas [5, 46]. The reason mismatch repair deficient gliomas do not demonstrate the same degree of microsatellite instability as other cancer types is not well understood at present.

The nine patients (6 females, 3 males) with de novo RRD glioblastoma had a median age at diagnosis of 50 years (range 27–78 years). This was significantly younger than the other 450 patients with conventional IDH-wildtype glioblastomas lacking somatic hypermutation and mismatch repair deficiency who had a median age at diagnosis of 63 years (*p* < 0.01). Those patients with confirmed germline origin of the mismatch repair gene mutations (i.e. Lynch syndrome) were 27, 50, 57, and 62 years old at initial glioblastoma diagnosis, whereas those with exclusively somatic origin of the mismatch repair gene inactivation were 33, 40, and 49 years old at diagnosis. Thus, while the de novo RRD glioblastoma typically occurred at a younger age than conventional IDH-wildtype glioblastoma in this patient cohort, the younger age at diagnosis did not always correlate with underlying Lynch syndrome.

### Imaging features of “De novo replication repair deficient glioblastoma, IDH-wildtype”

**Fig. 1:**
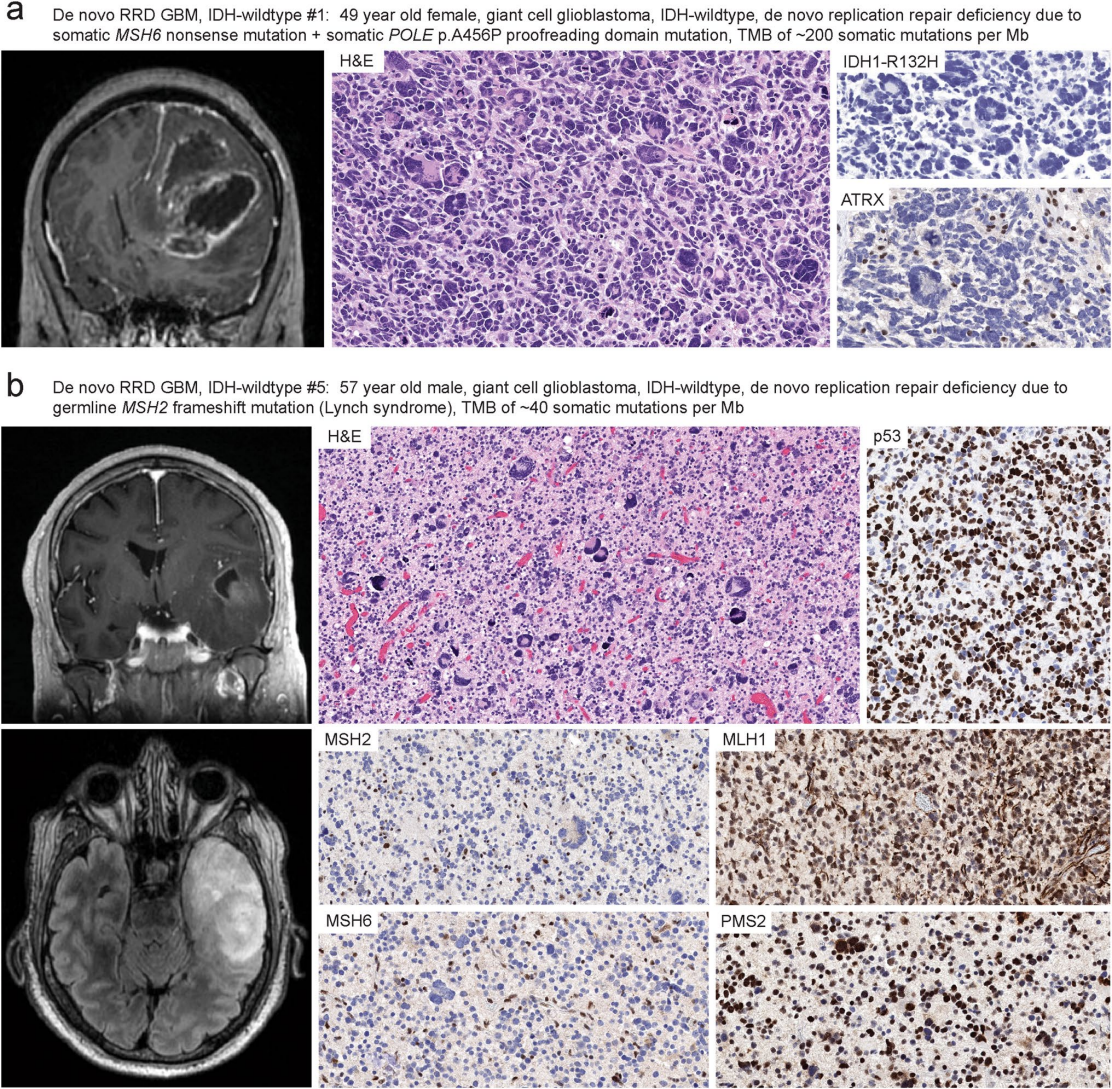
Imaging and histopathologic features of “De novo replication repair deficient glioblastoma, IDH-wildtype”. **a**, **b** Imaging features at initial presentation were indistinguishable from conventional glioblastoma, IDH-wildtype. All patients had mass lesions in the cerebral hemispheres demonstrating substantial mass effect, peripheral ring enhancement on post-contrast sequences, and extensive T2 FLAIR hyperintensity extending into the surrounding parenchyma reflective of the infiltrative growth patterns. The tumors were histologically composed of diffuse astrocytic gliomas with high cellularity, brisk mitotic activity, marked nuclear pleomorphism including frequent giant cells, microvascular proliferation, and palisading necrosis. All tumors were IDH-wildtype and negative for IDH1 p.R132H mutant protein expression by immunohistochemistry (**a**). Many tumors (5/9, 56%) contained inactivating *ATRX* mutations and had somatic loss of ATRX protein expression in tumor cells (**a**). Inactivating mutations in *TP53* were frequent in the tumor cohort (8/9, 89%), and those tumors with deleterious missense mutations demonstrated aberrant nuclear accumulation of p53 protein (**b**). All tumors demonstrated biallelic inactivation of a mismatch repair gene, which was either exclusively somatic or with one of the two events being present in the germline in the heterozygous state (Lynch syndrome) accompanied by somatic inactivation of the remaining allele. Immunohistochemistry demonstrated loss of expression of the affected mismatch repair protein in tumor cell nuclei (and concomitant MSH6 loss for those with *MSH2* mutational inactivation given the protein dimerization pattern of the mismatch repair complex), with retained/intact expression in endothelial cells and other admixed non-neoplastic cells (**b**). This is in contrast to children with constitutional mismatch repair deficiency (CMMRD) syndrome who have biallelic germline mutation in an MMR gene and whose tumors demonstrate loss of the affected MMR protein in both tumor cells and non-neoplastic cells (not shown, see references 5 and 9)

Pre-operative magnetic resonance imaging (MRI) studies of the nine patients with de novo RRD glioblastomas revealed characteristic features of glioblastoma without discernible radiologic hallmarks that enabled distinguishing from conventional IDH-wildtype glioblastomas lacking somatic hypermutation and mismatch repair deficiency. All patients had intraparenchymal mass lesions in the cerebral hemispheres demonstrating substantial mass effect, peripheral ring enhancement on post-contrast sequences, and extensive T2 FLAIR hyperintensity extending into the surrounding parenchyma reflective of the infiltrative growth patterns (Fig. 1, Supplementary Fig. S1, Supplementary Table S2). Three patients had tumors centered in the frontal lobes, three in the temporal lobes, one in the parietal lobes, one in the splenium of the corpus callosum, and one in the posterior third ventricle (pineal region). Two patients (#7 and #9) had evidence of dissemination throughout the ventricular system and along the spinal cord at initial presentation.

### Histopathologic features of “De novo replication repair deficient glioblastoma, IDH-wildtype”

The glioblastomas from these nine adult patients were all diffuse astrocytic gliomas with brisk mitotic activity, necrosis, and microvascular proliferation (Fig. 1). Each of the tumors contained numerous bizarre and multinucleated tumor giant cells, either focally or diffusely throughout the tumor, consistent with the giant cell histologic variant of glioblastoma. Immunohistochemistry revealed the tumors were uniformly negative for IDH1 p.R132H mutant protein expression, consistent with their underlying IDH-wildtype genotypes. Five of the nine tumors demonstrated somatic loss of ATRX protein expression that corresponded with those harboring inactivating *ATRX* mutations, which is known to be rare among IDH-wildtype glioblastomas in adults [31]. Aberrant accumulation of p53 protein was present in the majority of tumor nuclei in most tumors, corresponding with the high frequency of deleterious *TP53* missense mutations. Additionally, all tumors demonstrated somatic loss of expression in one of the four mismatch repair proteins (MSH2, MSH6, MLH1, or PMS2) resulting in mismatch repair deficiency. Immunohistochemistry revealed absence of the affected mismatch repair protein exclusively in tumor nuclei, with retained/intact expression in endothelial cells and other admixed non-neoplastic cells. This corresponded to the biallelic inactivation of the affected mismatch repair gene being either exclusively somatic in origin or due to a heterozygous germline mutation (Lynch syndrome) with somatic inactivation of the remaining allele. This is in contrast to children with constitutional mismatch repair deficiency (CMMRD) syndrome who have biallelic germline mutation in a mismatch repair gene and whose tumors demonstrate loss of the affected mismatch repair protein in both tumor cells and non-neoplastic cells [5, 9].

### Genomic landscape of “De novo replication repair deficient glioblastoma, IDH-wildtype”

**Fig. 2:**
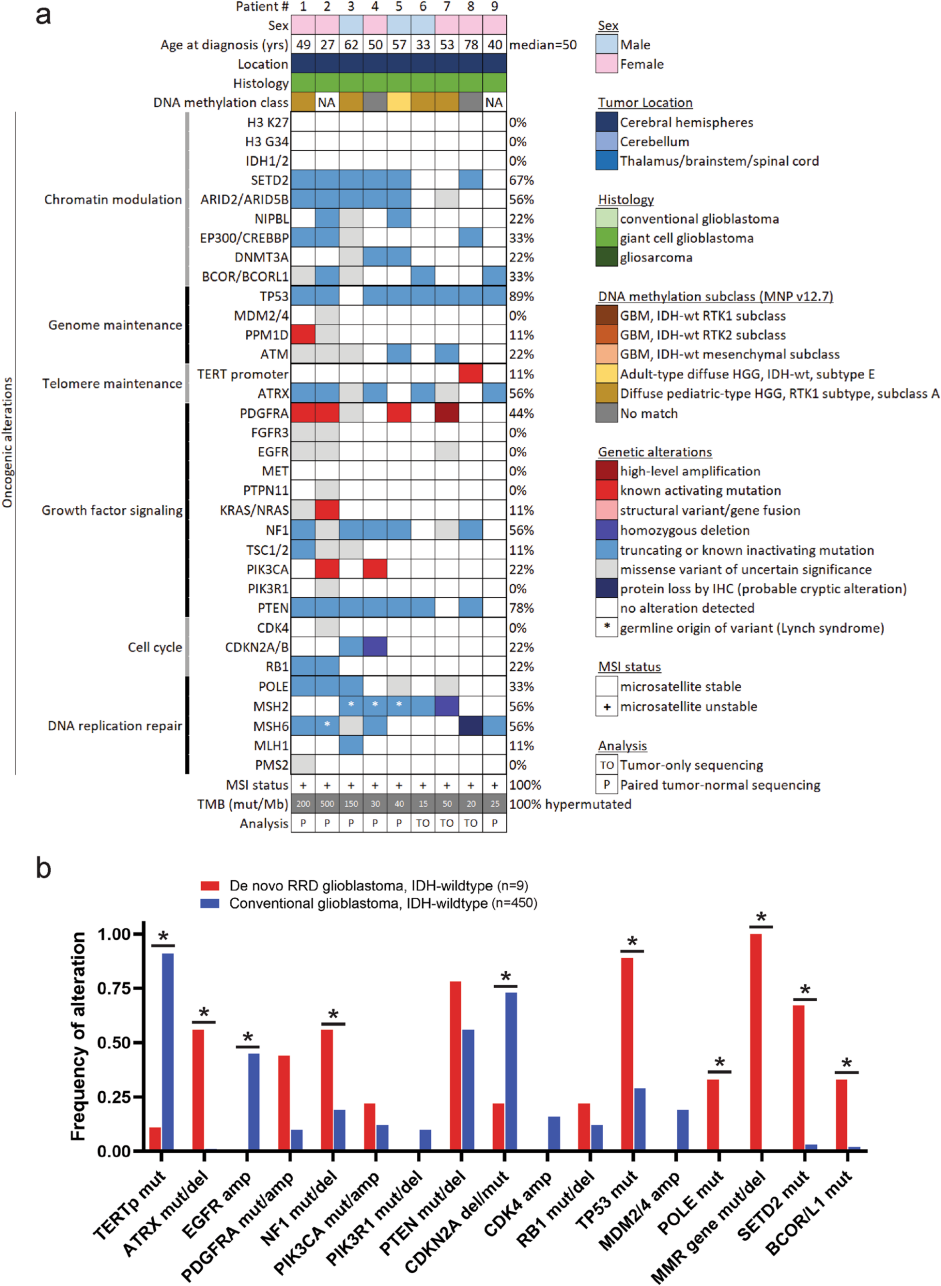
The genomic landscape of “De novo replication repair deficient glioblastoma, IDH-wildtype”. **a** Oncoprint summary plot of the clinical, histologic, genomic, and epigenomic features of the de novo RRD glioblastoma patient cohort. DNA methylation subclass assignment is based on random forest classification using version 12.7 of the DKFZ MolecularNeuropathology.org online classifier. See Supplementary Tables S3, S4, and S8 for source data. **b** Comparison of oncogenic alteration frequency between de novo RRD glioblastoma (*n* = 9) and conventional glioblastoma (*n* = 450) using the identical genomic testing platform and informatics pipeline. Significant differences in genetic alteration frequency are denoted with an asterisk (*p* < 0.05). See Table 2 and Supplementary Tables S3 and S4 for source data

Targeted next-generation DNA sequencing of the 9 de novo RRD glioblastomas revealed a unique genomic landscape compared to the other 450 conventional IDH-wildtype glioblastomas (Fig. 2, Table 2, Supplementary Tables S3 and S4). While telomere maintenance in the vast majority of glioblastomas is driven by one of two hotspot substitution mutations (c.-124C > T or c.-146C > T) in the promoter region of *TERT* [26], only one of the de novo RRD glioblastomas (1/9, 11%) had *TERT* promoter mutation or other *TERT* alteration, in contrast to 93% (418/450) of the 450 conventional glioblastomas. Instead, there were frequent inactivating mutations in *ATRX* known to cause alternative lengthening of telomeres (ALT) in five of the de novo RRD glioblastomas (5/9, 56%), which were rare in the conventional glioblastoma cohort (4/450, 1%). The most frequent oncogenic driver events in the de novo RRD glioblastomas were inactivating mutations in the *TP53* (8/9, 89%), *PTEN* (7/9, 78%), and *NF1* (5/9, 56%) tumor suppressor genes, which are known to be important glioblastoma tumor suppressors but were present at lower frequency in both our conventional IDH-wildtype glioblastoma cohort (*TP53*: 130/450, 29%; *PTEN*: 254/450, 56%; *NF1*: 84/450, 19%) and other prior glioblastoma datasets such as The Cancer Genome Atlas [10]. Notably, there was an absence of focal *EGFR* amplification in the de novo RRD glioblastomas (0/9, 0%), which was present in 45% (203/450) of the conventional glioblastomas. There was also an absence of *MET* amplification and *FGFR3* fusion in the de novo RRD glioblastomas. Instead, the receptor tyrosine kinase that was most often altered in the de novo RRD glioblastomas was *PDGFRA* (4/9, 44%), with three tumors harboring known activating missense mutations (p.D842Y, p.D561D, p.E229K) in the absence of *PDGFRA* gene amplification and a fourth tumor with low-level amplification of a mutant *PDGFRA* allele (p.D842V). While *PDGFRA* alterations were also present in a subset of the conventional glioblastomas (48/450, 11%), activating missense mutations in *PDGFRA* occurring in the absence of focal *PDGFRA* amplification were rare (2/450, < 1%), with the vast majority of conventional glioblastomas harboring focal high-level *PDGFRA* amplification (46 of the 48 altered tumors). In conventional IDH-wildtype glioblastomas, cell cycle dysregulation is most frequently achieved through *CDKN2A/B* homozygous deletion, *CDK4* amplification, or *RB1* mutation/deletion, which were present in 70% (316/450), 16% (71/450), and 12% (56/450) of our conventional glioblastoma cohort, respectively. Only one of the de novo RRD glioblastomas had *CDKN2A/B* homozygous deletion (1/9, 11%), with another tumor harboring a truncating frameshift mutation in *CDKN2A* that was a rare mechanism of *CDKN2A* gene inactivation in the conventional glioblastomas (11/450, 2%). None of the de novo RRD glioblastomas (0/9, 0%) had *CDK4* amplification, and two tumors had truncating mutations in *RB1* (2/9, 22%). Additionally, there were likely oncogenic truncating mutations recurrently affecting genes involved in histone tail methylation (*SETD2*: 6/9, 67%), histone tail acetylation (*EP300* or *CREBBP*: 3/9, 33%), chromatin remodeling (*ARID2* or *ARID5B*: 5/9, 56%), or BCL6-associated transcriptional co-repression (*BCOR* or *BCORL1*: 3/9, 33%) enriched in the de novo RRD glioblastomas compared to the conventional glioblastoma cohort.

**Table 2:** Comparison of oncogenic alteration frequency between "De novo replication repair deficient glioblastoma, IDH-wildtype" and conventional glioblastoma, IDH-wildtype

Gene	Oncogenic alteration	# of De novo RRD GBM with	Fraction of 9 De novo RRD GBM with	# of conventional GBM with	Fraction of 450 conventional GBM with	*p* value
H3 K27	H3F3A, H3F3B, HIST1H3B, or HIST1H3C p.K27 mutation	0	0.00	0	0.00	NA
H3 G34	H3F3A, H3F3B, HIST1H3B, or HIST1H3C p.G34 mutation	0	0.00	0	0.00	NA
IDH1/2	Any IDH1 p.R132 or IDH2 p.R172 mutation	0	0.00	0	0.00	NA
SETD2	Any likely oncogenic mutation or deletion	6	0.67	13	0.03	0
ARID2/ARID5B	Any likely oncogenic mutation or deletion	5	0.56	4	0.01	0
EP300/CREBBP	Any likely oncogenic mutation or deletion	3	0.33	4	0.01	3.77E−15
BCOR/BCORL1	Any likely oncogenic mutation or deletion	3	0.33	8	0.02	8.86E−10
TP53	Any likely oncogenic mutation or deletion	8	0.89	130	0.29	0.000102
MDM2/4	Amplification	0	0.00	86	0.19	0.145713
ATM	Any likely oncogenic mutation	2	0.22	3	0.01	6.89E−10
TERT promoter	c.-124C > T or c.-146C > T hotspot mutation	1	0.11	410	0.91	8.22E−15
TERT	Any likely oncogenic alteration including amplification or promoter rearrangement	1	0.11	418	0.93	0
ATRX	Any likely oncogenic mutation or deletion	5	0.56	4	0.01	0
PDGFRA	Amplification	1	0.11	46	0.10	0.930598
PDGFRA	Any likely oncogenic mutation occurring in absence of amplification	3	0.33	2	0.00	0
FGFR3	Fusion	0	0.00	8	0.02	0.686557
EGFR	Amplification	0	0.00	203	0.45	0.006975
EGFR	Any likely oncogenic alteration including amplification, mutation, intragenic deletion, fusion, or rearrangement	0	0.00	226	0.50	0.002845
MET	Amplification or fusion	0	0.00	15	0.03	0.577597
PTPN11	Any likely oncogenic mutation	0	0.00	12	0.03	0.619591
KRAS/NRAS	Amplification or any likely oncogenic mutation	1	0.11	9	0.02	0.063756
NF1	Any likely oncogenic mutation, deletion, or rearrangement	5	0.56	84	0.19	0.005578
TSC1/2	Any likely oncogenic mutation or deletion	1	0.11	10	0.02	0.084272
PIK3CA	Any likely oncogenic mutation or amplification	2	0.22	54	0.12	0.353535
PIK3R1	Any likely oncogenic mutation or deletion	0	0.00	43	0.10	0.330001
PTEN	Any likely oncogenic mutation or deletion	7	0.78	254	0.56	0.200722
CDK4	Amplification	0	0.00	71	0.16	0.194945
CDKN2A	Homozygous deletion or any likely oncogenic mutation	2	0.22	327	0.73	0.000882
RB1	Any likely oncogenic mutation or deletion	2	0.22	56	0.12	0.382035
POLE	Any pathogenic proofreading domain mutation	3	0.33	0	0.00	0
MMR genes	Any pathogenic MSH2, MSH6, MLH1, or PMS2 mutation or deletion	9	1.00	2	0.00	0

**Fig. 3:**
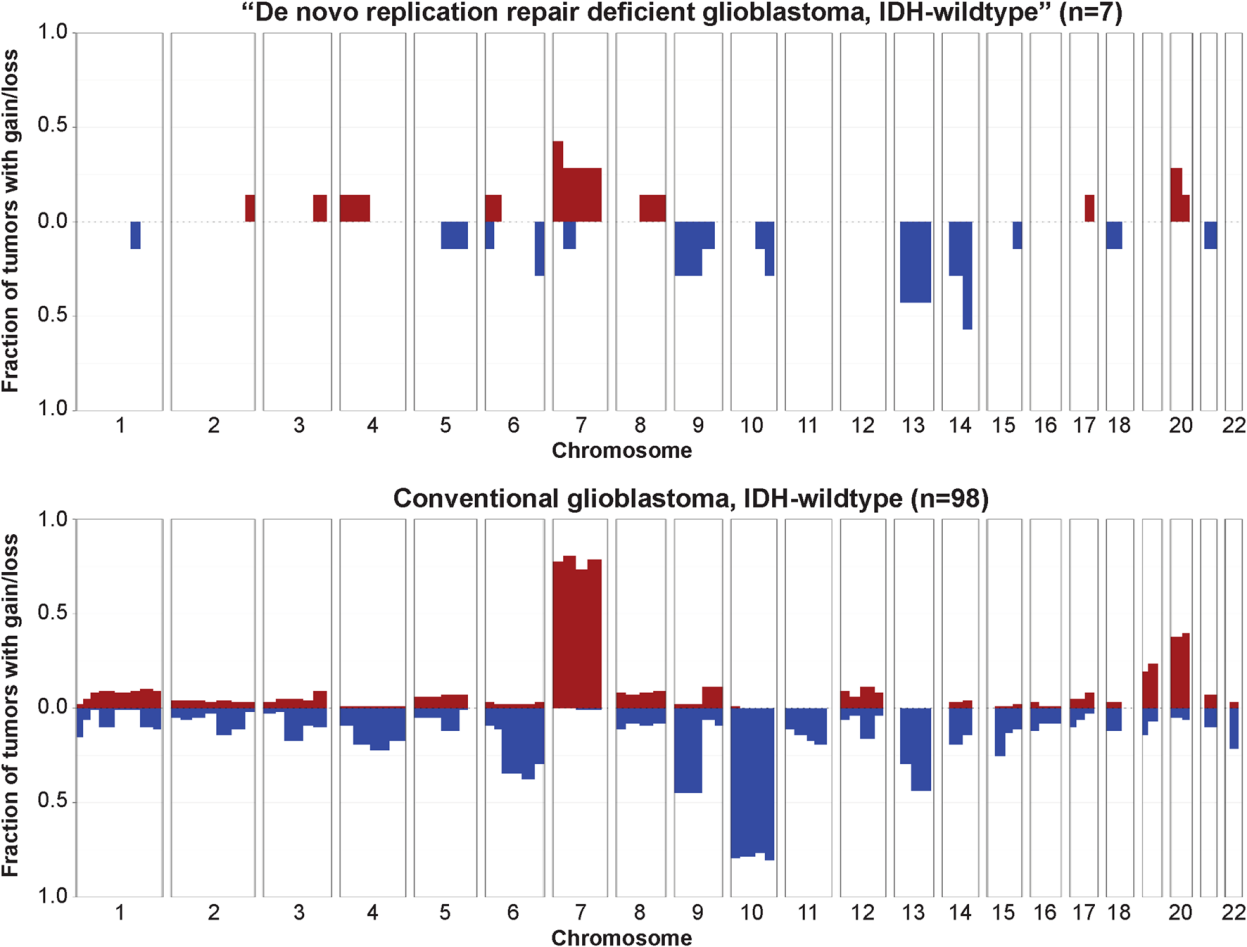
“De novo replication repair deficient glioblastoma, IDH-wildtype” lacks many of the recurrent chromosomal copy number alterations present in conventional glioblastoma, IDH-wildtype including trisomy/gain of chromosome 7 and monosomy/loss of chromosome 10. Shown are copy number summary plots illustrating the fraction of tumors with gain or loss along each chromosome for 7 de novo RRD glioblastomas and 98 conventional glioblastomas. See Supplementary Table S4 for source data and Supplementary Fig. S2 for representative copy number plots of individual tumors

Chromosomal copy number analysis of the 9 de novo RRD glioblastomas revealed an absence of the combined trisomy/gain of chromosome 7 and monosomy/loss of chromosome 10 that is typical of conventional glioblastoma, IDH-wildtype (Fig. 3, Supplementary Fig. S2, Supplementary Table S4). Most tumors displayed only a few chromosomal gains or losses per tumor with few, if any, focal amplifications or deep deletions. Only one tumor (1/9, 11%) demonstrated focal amplification of *PDGFRA* on chromosome 4q12, and only one tumor (1/9, 11%) demonstrated focal homozygous/biallelic deletion of *CDKN2A* and *CDKN2B* on chromosome 9p21. No tumors demonstrated *EGFR* amplification, *MET* amplification, *CDK4* amplification, *MDM2* or *MDM4* amplification, *PTEN* homozygous deletion, or *NF1* homozygous deletion that are frequent oncogenic events in conventional IDH-wildtype glioblastomas. The latter two genes (*PTEN* and *NF1*) were frequently inactivated in the de novo RRD glioblastomas through short somatic variants as described above, but larger gene deletions were not present in this tumor cohort. Also notably, two of the nine (22%) de novo RRD glioblastomas (patients #2 and #9) demonstrated near genomic haploidization and subsequent reduplication of most chromosomes in the genome, as was recently reported in a unique molecular subset of giant cell glioblastomas [4]. This near genomic haploidization may likely have served as the tumor-initiating event by eliminating the remaining *MSH6* wildtype allele, thereby resulting in the mismatch repair deficiency that led to accumulation of mutations in oncogenes and tumor suppressor genes that drove tumor development. Altogether, the de novo RRD glioblastomas had a distinct genomic landscape compared to the other 450 conventional IDH-wildtype glioblastomas, with a unique spectrum of oncogenic driver events that were predominantly short somatic mutations (single nucleotide substitutions or small insertions/deletions) targeting *TP53*, *PTEN*, *NF1*, *ATRX*, *SETD2*, and *PDGFRA*, along with a paucity of focal amplification and gene deletion events that are frequent in conventional IDH-wildtype glioblastoma (e.g.,* EGFR* amplification, *CDKN2A/B* homozygous deletion, *CDK4* amplification, *MDM2* and *MDM4* amplification).

### Unique DNA methylation profiles of “De novo replication repair deficient glioblastoma, IDH-wildtype”

**Fig. 4:**
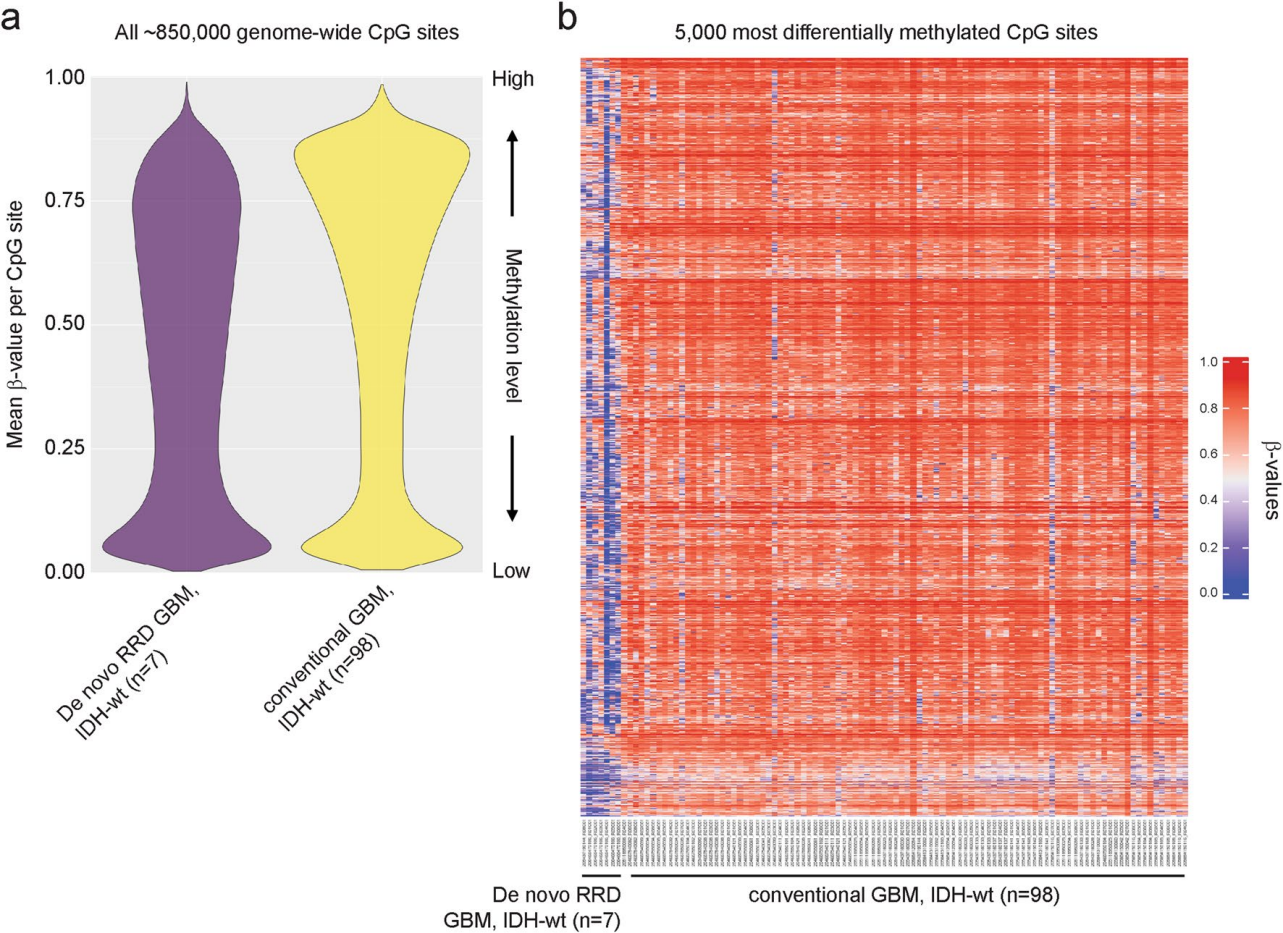
“De novo replication repair deficient glioblastoma, IDH-wildtype” has a distinct global hypomethylation epigenetic signature compared to conventional glioblastoma, IDH-wildtype. **a** Violin plot of DNA methylation data showing the mean beta-value for each of approximately 850,000 CpG sites across 7 de novo RRD glioblastomas and 98 conventional glioblastomas. **b** Heatmap of DNA methylation profiles for 7 de novo RRD glioblastoma alongside 98 conventional glioblastoma. Shown are the 5000 most differentially methylated probes amongst the 105 glioblastomas revealing extensive hypomethylation of CpG sites in the de novo RRD glioblastoma compared to the cohort of conventional glioblastomas. See Supplementary Table S5 for detailed annotations of these 5000 most differentially methylated CpG sites

**Fig. 5:**
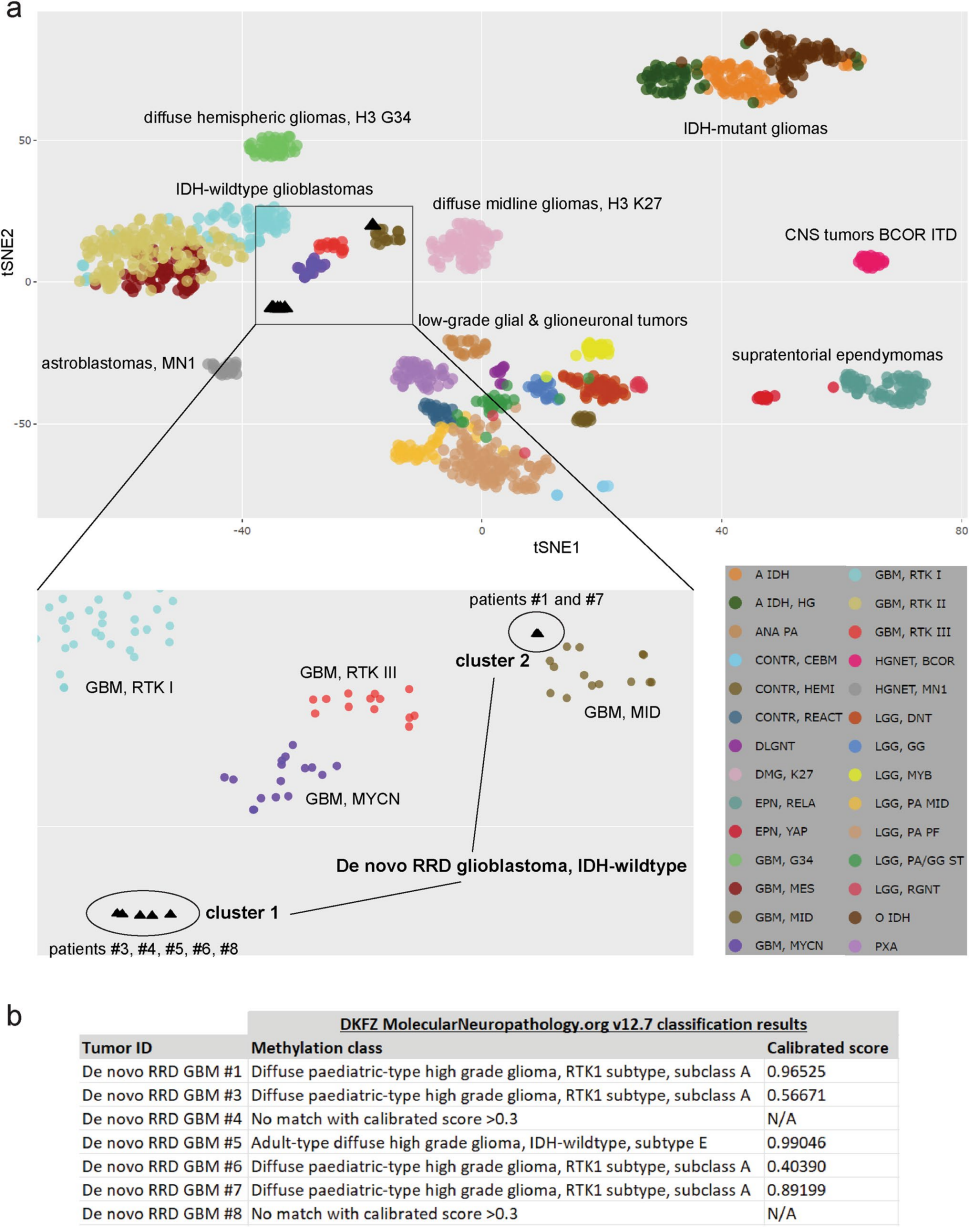
“De novo replication repair deficient glioblastoma, IDH-wildtype” has a unique epigenetic signature distinct from all established reference methylation classes of IDH-wildtype glioblastomas in adults. **a** tSNE dimensionality reduction plot of genome-wide DNA methylation profiles for 7 de novo RRD glioblastomas alongside 1143 reference samples spanning 25 CNS tumor methylation groups and 3 control tissue methylation groups. See Supplementary Table S7 for sample manifest. **b** Results of DNA methylation-based classification for the 7 de novo RRD glioblastoma samples using version 12.7 of the DKFZ Molecular Neuropathology classifier. See Supplementary Table S8 for further details

Genome-wide DNA methylation profiling was performed on a subset of the de novo RRD glioblastomas (*n* = 7) and a subset of the conventional IDH-wildtype glioblastomas (*n* = 98) to study their epigenomes. We found that the de novo RRD glioblastomas had unique hypomethylated genomes in comparison to the conventional IDH-wildtype glioblastomas, with several thousand recurrently hypomethylated CpG sites shared across the 7 de novo RRD glioblastomas versus the conventional glioblastomas (Fig. 4, Supplementary Table S5). KEGG pathway analysis of the genes containing the 5000 most differentially methylated CpG sites that were hypomethylated in de novo RRD glioblastoma compared to conventional glioblastomas revealed enrichment for specific biologic processes including inositol phosphate metabolism, mRNA surveillance, and neurotrophin signaling pathway (Supplementary Table S6). tSNE dimensionality reduction of DNA methylation profiles from the 7 de novo RRD glioblastomas together with 1143 reference samples spanning 25 CNS tumor methylation groups and 3 control tissue methylation groups demonstrated two clusters of de novo RRD glioblastomas that were distinct from all established adult-type IDH-wildtype glioblastoma reference classes (Fig. 5a, Supplementary Table S7). Cluster 1 was composed of de novo RRD glioblastomas from five patients (#3, #4, #5, #6, and #8), while cluster 2 was composed of de novo RRD glioblastomas from two patients (#1 and #7). No underlying differences in patient sex, age at diagnosis, tumor location, presence/absence of *POLE* mutation, or somatic versus germline origin of mismatch repair gene mutation were appreciable between the two DNA methylation clusters of de novo RRD glioblastomas. Next, random forest classification of the DNA methylation profiles was performed to compare each tumor’s epigenetic signature against established reference methylation classes of CNS tumors as previously described using the DKFZ MolecularNeuropathology.org online classifier (v.12.7) [14]. None of the evaluated tumors aligned with the established adult-type IDH-wildtype glioblastoma reference classes, but instead mostly classified as “Diffuse pediatric-type high grade glioma, RTK1 subtype, subclass A (novel)” (Fig. 5b, Supplementary Table S8). This is a new provisional reference class added to version 12.7 of the DKFZ Molecular Neuropathology classifier that is not yet thoroughly characterized, but is the methylation subclass where CMMRD-associated diffuse pediatric-type high-grade gliomas are known to reside [19]. Notably, one tumor aligned with high calibrated score to another novel uncharacterized methylation class “Adult-type diffuse high-grade glioma, IDH-wildtype, subtype E”, and two others did not match with any reference classes in the current version of the classifier. Together, these findings indicate that de novo RRD glioblastomas have a unique hypomethylated epigenetic signature compared to established adult IDH-wildtype glioblastoma reference classes, with closest similarity to a novel subclass of diffuse pediatric-type high-grade gliomas that includes those arising in the setting of CMMRD, but may be epigenetically heterogeneous.

### Rare conventional IDH-wildtype glioblastomas contain heterozygous MMR gene mutations without underlying replication repair deficiency

Among the 450 primary treatment-naïve conventional IDH-wildtype glioblastomas lacking somatic hypermutation, two tumors (2/450, < 1%) contained heterozygous inactivating mutations in a canonical mismatch repair gene affecting one of two alleles (Supplementary Fig. S3). Both were *MSH6* frameshift mutations (p.A61fs and p.Y397fs), one of which was confirmed to be somatic and the other had uncertain somatic versus germline origin due to tumor-only sequencing analysis. No inactivation of the remaining *MSH6* wildtype allele was identified, and both tumors demonstrated retained/intact expression of MSH6 protein in tumor nuclei by immunohistochemistry. Both of these glioblastomas with heterozygous *MSH6* mutations had low somatic mutation burden with TMB values of less than 5 mutations per Mb, and both were microsatellite stable with instability at less than 2% of the 86 evaluated microsatellites. Both tumors demonstrated typical glioblastoma histology with absence of giant cell morphology. These two tumors had the combination of trisomy chromosome 7 and monosomy chromosome 10 along with genetic alterations typical of conventional IDH-wildtype glioblastoma (e.g.,* TERT* promoter mutation, *CDKN2A/B* homozygous deletion, *EGFR* amplification). Furthermore, both tumors had DNA methylation profiles that aligned with reference methylation classes of adult-type glioblastoma, IDH-wildtype with high calibrated scores. These findings indicate that rare conventional IDH-wildtype glioblastomas in adults can acquire heterozygous mutations in MMR genes during tumor development that do not correspond with underlying mismatch repair deficiency and otherwise fail to align with the characteristic features of “De novo replication repair deficient glioblastoma, IDH-wildtype”.

### Distinct cellular composition of “De novo replication repair deficient glioblastoma, IDH-wildtype”

**Fig. 6:**
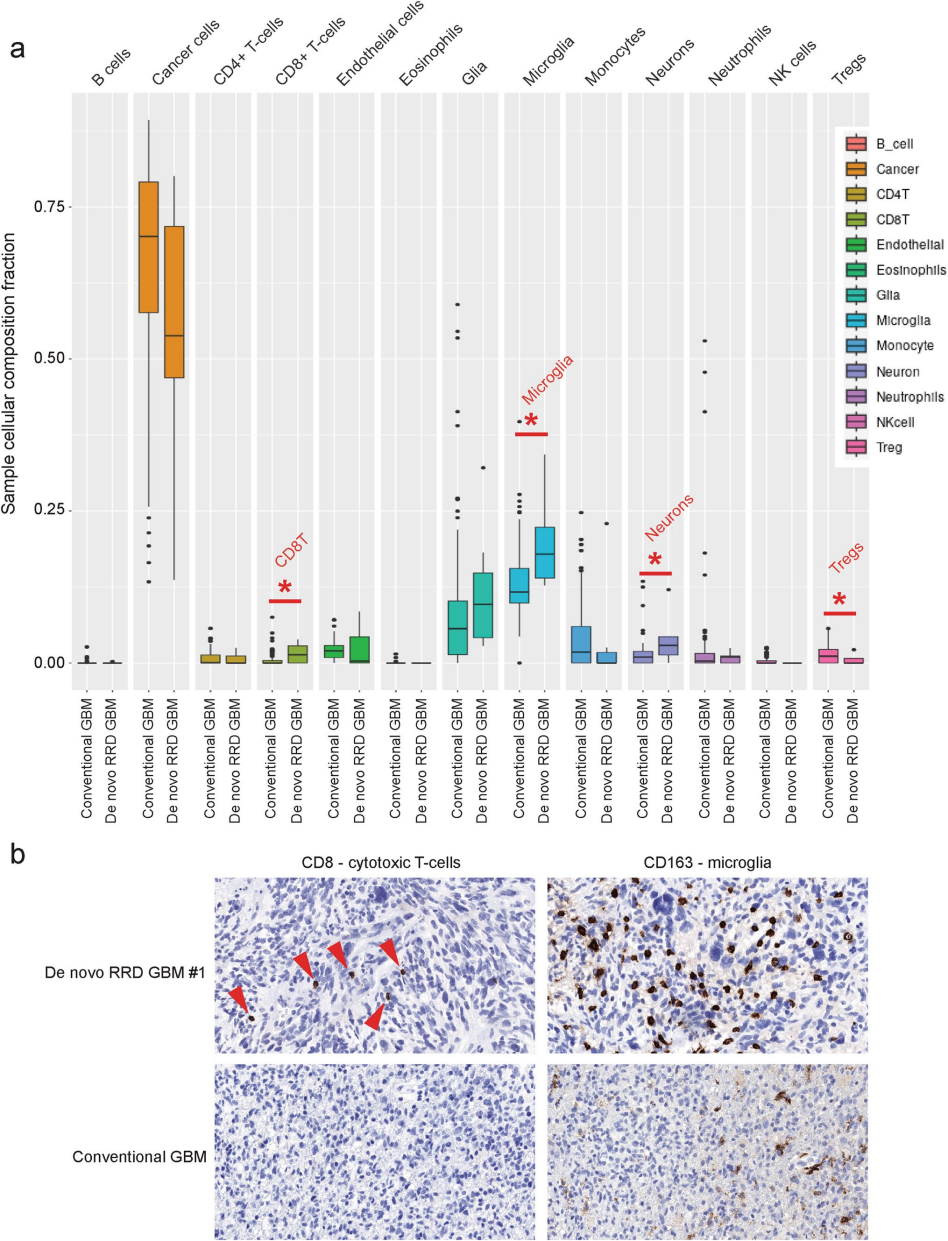
Deconvolution analysis reveals a unique cellular composition of “De novo replication repair deficient glioblastoma, IDH-wildtype” compared to conventional glioblastoma, IDH-wildtype. **a** Cellular composition of 7 de novo RRD glioblastoma and 98 conventional glioblastoma was estimated by methyCIBERSORT deconvolution of Infinium EPIC DNA methylation profiles at time of initial diagnostic surgery before any adjuvant therapy or immune checkpoint blockade. The de novo RRD glioblastomas demonstrated a greater proportion of microglia and CD8 + T-cells, and lower proportion of regulatory T-cells (Tregs) compared to conventional glioblastoma. See Supplementary Table S9 for source data. **b** Representative photomicrographs of immunohistochemistry for CD8 and CD163 on a de novo RRD glioblastoma and a conventional glioblastoma for comparison

Deconvolution of DNA methylation array data was performed with methylCIBERSORT using a custom CNS tumor signature matrix to estimate neoplastic cell and various tumor microenvironment cell fractions for 7 de novo RRD glioblastomas and 98 conventional IDH-wildtype glioblastomas, all of which were initial primary surgical resection specimens prior to any radiation, chemotherapy, or immunotherapy agents. We found that the de novo RRD glioblastomas were significantly enriched in microglia and CD8 + T-cells and had fewer regulatory T-cells (Tregs) compared to the conventional glioblastomas while having relatively similar tumor cell fractions (Fig. 6a, Supplementary Table S9). Immunohistochemistry performed on a subset of the tumors demonstrated a population of intratumoral CD8 + T-lymphocytes in the de novo RRD glioblastomas that was minimal to absent in the evaluated conventional IDH-wildtype glioblastomas (Fig. 6b). These CD8 + T-lymphocytes were interspersed among the tumor cells and were not accompanied by a significant population of CD4 + T-lymphocytes. We also observed a robust number of intratumoral microglia highlighted by CD68 and CD163 immunostaining in de novo RRD glioblastomas. This indicates that de novo RRD glioblastomas may have a distinct immune cell microenvironment compared to conventional glioblastomas, perhaps due to the somatic hypermutation and substantially increased neoantigen load on tumor cells expected as a result, which could potentially make them more susceptible to immune cell clearance following immune checkpoint blockade.

### Prolonged survival of patients with “De novo replication repair deficient glioblastoma, IDH-wildtype” and potential benefit from immune checkpoint blockade

**Fig. 7:**
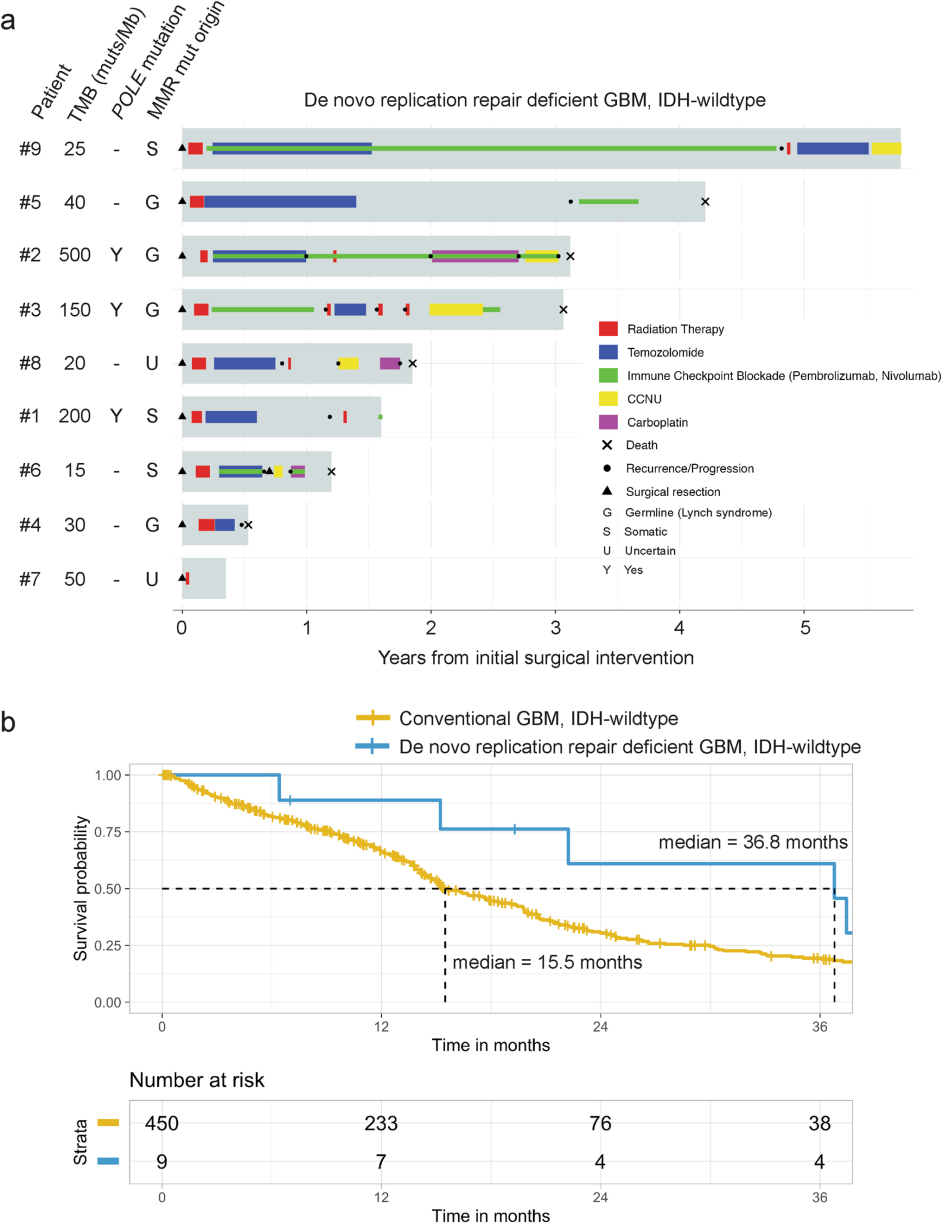
Patients with “de novo replication repair deficient glioblastoma, IDH-wildtype” have prolonged survival compared to conventional glioblastoma, IDH-wildtype and may benefit from immune checkpoint blockade. **a** Swimmer’s plot showing timing of initial surgical intervention, radiation, chemotherapy, immune checkpoint blockade with either pembrolizumab or nivolumab, and clinical outcomes for the 9 patients with de novo replication repair deficient glioblastoma, IDH-wildtype. See Supplementary Table S1 for further clinical data including extent of resection and treatment regimen. **b** Kaplan–Meier curves showing overall survival for the 459 consecutive adult patients with IDH-wildtype glioblastoma in the cerebral hemispheres, stratified by those with “de novo replication repair deficient glioblastoma, IDH-wildtype” (*n* = 9, median survival 36.8 months) versus those with conventional glioblastoma (*n* = 450, median survival 15.5 months)

Given that de novo RRD glioblastomas have a unique genomic and epigenomic landscape and immune cell microenvironment, we therefore sought to determine the clinical outcomes for affected patients and speculated that it might be different than conventional IDH-wildtype glioblastoma. Detailed clinical data on extent of resection, adjuvant treatment regimens, time to progression, salvage therapy, and overall survival are presented in Supplementary Table S1. Given the prospective clinical genomic testing that identified somatic hypermutation and mismatch repair deficiency at the time of initial surgical intervention, five of the nine patients were treated off-label with immune checkpoint blockade (either pembrolizumab or nivolumab) during their treatment course (Fig. 7). A sixth patient (#1) was just beginning pembrolizumab at the time of manuscript drafting. Patients #2, #3, #6, and #9 were treated with immune checkpoint blockade in the adjuvant setting immediately following completion of radiation—patient #3 received nivolumab alone without concurrent temozolomide, while patients #2, #6, and #9 received adjuvant temozolomide with concurrent immune checkpoint blockade. At time of recurrence/progression, patient #5 who hadn’t received immune checkpoint blockade therapy in the adjuvant setting was then treated with pembrolizumab. Patients #2, #3, and #6, who received immune checkpoint blockade in the adjuvant setting, were also given additional immune checkpoint blockade therapy at the time of recurrence/progression. The survival times for the five patients whose treatment included immune checkpoint blockade were 37.4, 36.8, 50.5, 15.2, and 69.4 months. The median survival for the total cohort of nine patients with de novo RRD glioblastoma was 36.8 months (95% confidence interval: 22-not reached), which was significantly longer than the other 450 adult patients with conventional IDH-wildtype glioblastoma lacking somatic hypermutation and mismatch repair deficiency (median survival of 15.5 months, 95% confidence interval: 14.7–17.9, *p* < 0.001).

## Discussion

Through prospective genomic profiling of primary de novo IDH-wildtype glioblastomas in a large cohort of adults, we have identified a new distinct glioblastoma subtype that appears to be unique in its age at presentation, histological features, oncogenic driver events, mechanism of telomere maintenance, chromosomal copy number profiles, DNA methylation patterns, cellular composition, and clinical outcomes. We have provisionally termed this new subtype “De novo replication repair deficient glioblastoma, IDH-wildtype”, onto which “arising in the setting of genetically confirmed (or clinically diagnosed) Lynch syndrome” could be added to this integrated diagnosis as appropriate. While this subtype is rare and accounts for approximately 2% of all IDH-wildtype glioblastomas based on the current 2021 WHO Classification definition, we believe that it warrants recognition as a distinct subtype moving forward given both the unique underlying biology and the more favorable prognosis with potential benefit from immune checkpoint blockade suggested by this study and prior case reports [7, 20, 24].

The definitional criteria we propose for this unique subtype is a de novo/primary treatment-naïve IDH-wildtype and histone H3-wildtype glioblastoma occurring in an adult with next-generation DNA sequencing demonstrating somatic hypermutation and biallelic inactivation of a canonical mismatch repair gene (*MSH2*, *MSH6*, *MLH1*, or *PMS2*) via gene deletion, truncating mutation, or known pathogenic missense mutation. When feasible, examination of the mutational signature should demonstrate a signature consistent with mismatch repair deficiency and also *POLE* proofreading domain deficiency for those with accompanying *POLE* mutation. Microsatellite stability testing may reveal a low to moderate level of microsatellite instability or may be stable by some assays [5, 46]. Follow-up immunohistochemistry demonstrating loss of the affected mismatch repair protein in tumor nuclei with retained expression in endothelial and other non-neoplastic cells is recommended when feasible. Suspicion for this glioblastoma subtype should arise in patients with a known personal or family history of Lynch syndrome or early-onset colorectal cancer, as well as other Lynch syndrome-related tumor types including endometrial carcinoma, upper urothelial tract carcinoma, and sebaceous neoplasms. Histology revealing the giant cell variant of glioblastoma should also prompt consideration of this subtype. However, while all of the de novo RRD glioblastomas in this cohort demonstrated abundant giant cells either focally or diffusely throughout the tumor, not all “giant cell glioblastomas” have replication repair deficiency and belong to this subtype. *ATRX* gene mutation or ATRX protein deficiency by immunohistochemistry in an IDH-wildtype glioblastoma should also raise suspicion diagnostically for this subtype. DNA methylation profiling of an IDH-wildtype glioblastoma in an adult that classifies as “Diffuse pediatric-type high grade glioma, RTK1 subtype, subclass A” using version 12.7 of the DKFZ Molecular Neuropathology classifier may be another indication to consider and evaluate for this subtype.

One important exclusionary criterion for this subtype is the presence of an oncogenic IDH mutation, specifically *IDH1* p.R132 or *IDH2* p.R172 variants. Given the very high somatic mutation burden in hypermutant gliomas, many genes in the genome are riddled with single nucleotide substitutions and small indels. The majority of these are bystander or passenger variants resulting from the replication repair deficiency and are not tumorigenic/oncogenic, which can include variants in the *IDH1* and *IDH2* genes other than the known oncogenic p.R132 and p.R172 hotspots that are likely non-functional and not tumorigenic. However, there are true primary de novo IDH-mutant astrocytomas with both *IDH1* p.R132 mutations and biallelic inactivation of a canonical mismatch repair gene with associated somatic hypermutation—these tumors form a distinct epigenetic cluster and represent a poor prognostic subtype of IDH-mutant astrocytoma termed “Primary mismatch repair deficient astrocytoma, IDH-mutant” [45]. Similar to “De novo replication repair deficient glioblastoma, IDH-wildtype”, many (but not all) patients with primary mismatch repair deficient IDH-mutant astrocytomas have underlying Lynch syndrome with a heterozygous germline mutation in one of the mismatch repair genes and somatic inactivation of the remaining allele. Most patients are in their teenage years or 20’s at time of diagnosis for these primary mismatch repair deficient IDH-mutant astrocytomas. It remains an outstanding question why some Lynch syndrome patients develop IDH-mutant astrocytomas during the second to third decades of life, while others develop IDH-wildtype glioblastomas during the third to seventh decades. Furthermore, the biologic basis of the discrepancy in clinical outcomes with the mismatch repair deficiency conferring worse survival in primary IDH-mutant astrocytomas versus more favorable survival in primary IDH-wildtype glioblastomas compared to their respective non-hypermutant counterparts remains uncertain.

A second important exclusionary criterion for this subtype is somatic hypermutation and mismatch repair deficiency that is acquired at recurrence following treatment with temozolomide and did not exist in the primary treatment-naïve glioblastoma. Chemotherapy with the alkylating agent temozolomide is known to cause selection for mismatch repair inactivation and somatic hypermutation in recurrent gliomas that initially were mismatch repair intact with low somatic mutation burden as is typical for the vast majority of sporadic/spontaneous gliomas [25, 46, 48]. This phenomenon of acquired somatic hypermutation following temozolomide treatment has been documented in several glioma types including IDH-mutant astrocytomas, IDH-mutant and 1p/19q-codeleted oligodendrogliomas, IDH-wildtype glioblastomas, and histone H3 G34-mutant diffuse hemispheric gliomas [12, 41, 46, 49, 50]. This temozolomide-induced hypermutation has a distinct mutational signature characterized virtually exclusively by C > T:G > A transitions [1, 12]. This is different than the mutational signature caused by primary mismatch repair deficiency, which also features C > A:G > T transversions and short insertions/deletions at mononucleotide and polynucleotide repeats (often resulting in reading frameshifts), in addition to an abundance C > T:G > A transitions [1, 12]. Notably, these gliomas with secondary mismatch repair deficiency and somatic hypermutation acquired in response to treatment with temozolomide have a different underlying biology than primary de novo gliomas arising due to mismatch repair deficiency as the initiating oncogenic mechanism responsible for causing the genetic driver events that fueled tumor development. For example, we do not believe that an IDH-wildtype glioblastoma aligning with the RTK2 methylation class of adult glioblastomas that has *TERT* promoter mutation, *CDKN2A/B* homozygous deletion, *EGFR* amplification, and combined trisomy 7 plus monosomy 10 with low tumor mutation burden at time of initial resection which acquires hypermutation and mismatch repair deficiency at time of recurrence/progression should be considered equivalent to the de novo RRD glioblastomas described in this study. Not only are there different oncogenic driver events and epigenetic states, but there may also likely be a different cell of origin and/or a different response to therapy including immune checkpoint blockade between these two glioblastomas despite sharing somatic hypermutation and mismatch repair deficiency.

Notably, we identified that IDH-wildtype glioblastomas arising in the setting of Lynch syndrome can occur into adulthood as late as the 6th and 7th decades of life. While this tumor subtype occurred at a younger age than conventional IDH-wildtype glioblastoma in our cohort, the younger age at diagnosis did not always correlate with underlying Lynch syndrome. Multiple young adults with primary mismatch repair deficient IDH-wildtype glioblastoma in our cohort (ages 33, 40, and 49 years) had exclusively somatic inactivation of the mismatch repair gene. We believe that identifying patients with this glioblastoma subtype (no matter the specific age at diagnosis) should prompt genetic counseling and germline testing to evaluate for underlying Lynch syndrome, which could enable life-saving prospective cancer surveillance for those patients and family members with confirmed Lynch syndrome.

While we have provisionally termed this new diffuse glioma subtype occurring in adults as “De novo replication repair deficient glioblastoma, IDH-wildtype”, there are alternative classification options to consider and resolve through future studies. First, while we have proposed this new group of tumors as a unified subtype, we recognize there still exists heterogeneity among this subtype in terms of: (a) germline vs. somatic origin of the underlying MMR gene mutation, (b) presence vs. absence of accompanying *POLE* mutation and associated ultrahypermutation, and (c) diverse epigenetic states with at least two distinct clusters observed in our tSNE plot of DNA methylation profiles and alignment with multiple different reference classes by random forest classification. Second, while we have proposed this new group of tumors as a subtype (variant) of IDH-wildtype glioblastoma, an alternative consideration is that these tumors should be considered a new distinct tumor type (entity) separate from glioblastoma. A different taxonomy to consider might be a new glioma family termed “De novo replication repair deficient high-grade glioma”, with unique types being the IDH-wildtype tumors occurring in adults as described in this study (mixture of sporadic and Lynch-associated), the IDH-mutant tumors occurring in adolescents and young adults (mostly Lynch-associated), and the IDH- and H3-wildtype tumors occurring in children (mostly CMMRD-associated).

In summary, our multiplatform molecular analysis has provided further substantial evidence that “De novo replication repair deficient glioblastoma, IDH-wildtype” should be regarded as a distinct subtype of IDH-wildtype glioblastoma in the adult population. These tumors generally occur at a younger age compared to conventional glioblastomas and mostly lack the molecular hallmarks of conventional glioblastoma including *EGFR* amplification, trisomy 7 plus monosomy 10, *TERT* promoter mutation, and *CDKN2A/B* homozygous deletion. Instead, they are driven by genetic instability and harbor frequent inactivating mutations in *TP53*, *NF1*, *PTEN*, *ATRX*, and *SETD2* and recurrent activating mutations in *PDGFRA*, with a paucity of focal amplifications and deep deletions. They have unique hypomethylated epigenomes with greatest similarity to a novel subclass of diffuse pediatric-type high grade gliomas. Together with a few prior case reports of *POLE* mutant and mismatch repair deficient IDH-wildtype glioblastomas with favorable clinical course and response to immune checkpoint blockade [7, 20, 24], our patient cohort further suggests that de novo RRD glioblastomas may have better clinical outcomes than conventional glioblastoma overall and potentially benefit from immune checkpoint blockade. Further study is warranted to investigate the natural history of these tumors and more conclusively determine whether the prolonged survival many of these patients have experienced is due to their underlying biologic differences or is specifically attributable to the immune checkpoint blockade that some patients have been treated with. As acquired mismatch repair deficiency may underlie a treatment resistance mechanism to the alkylating effects of temozolomide [25, 48, 50], whether or not patients with de novo RRD glioblastoma derive clinical benefit from temozolomide remains uncertain, and the potential efficacy of chemotherapy agents with other mechanisms of action (i.e. lomustine [CCNU]) in this patient population remains to be explored. Nonetheless, we conclude that “De novo replication repair deficient glioblastoma, IDH-wildtype” represents a biologically distinct subtype in the adult population that will benefit from prospective identification and unique treatment, potentially including immune checkpoint blockade.

### Supplementary Information

Below is the link to the electronic supplementary material.

Supplementary file 1 (PDF 3056 KB)

Supplementary file 2 (XLSX 1139 KB)

## Data Availability

Raw and processed DNA methylation data from the de novo RRD glioblastoma cohort have been deposited at the NCBI Gene Expression Omnibus (GEO) under accession number GSE239715. Digitally scanned image files of representative H&E and immunostained sections from the de novo RRD glioblastomas are available at the following link: https://figshare.com/projects/De_novo_replication_repair_deficient_glioblastoma_IDH-wildtype/176784. Annotated DNA sequencing data from the de novo RRD glioblastoma cohort are provided in the supplementary data tables. Raw sequencing data files are available from the authors upon request.
