# Supplementary material for: “De novo replication repair deficient glioblastoma, IDH-wildtype” is a distinct glioblastoma subtype in adults that may benefit from immune checkpoint blockade
Source: Acta Neuropathol. 2023 Dec 11;147(1):3. doi: 10.1007/s00401-023-02654-1 (PMC10713691; doi:10.1007/s00401-023-02654-1)
Supplement: Supplementary file 1 — (PDF 3056 KB) [file 401_2023_2654_MOESM1_ESM.pdf]

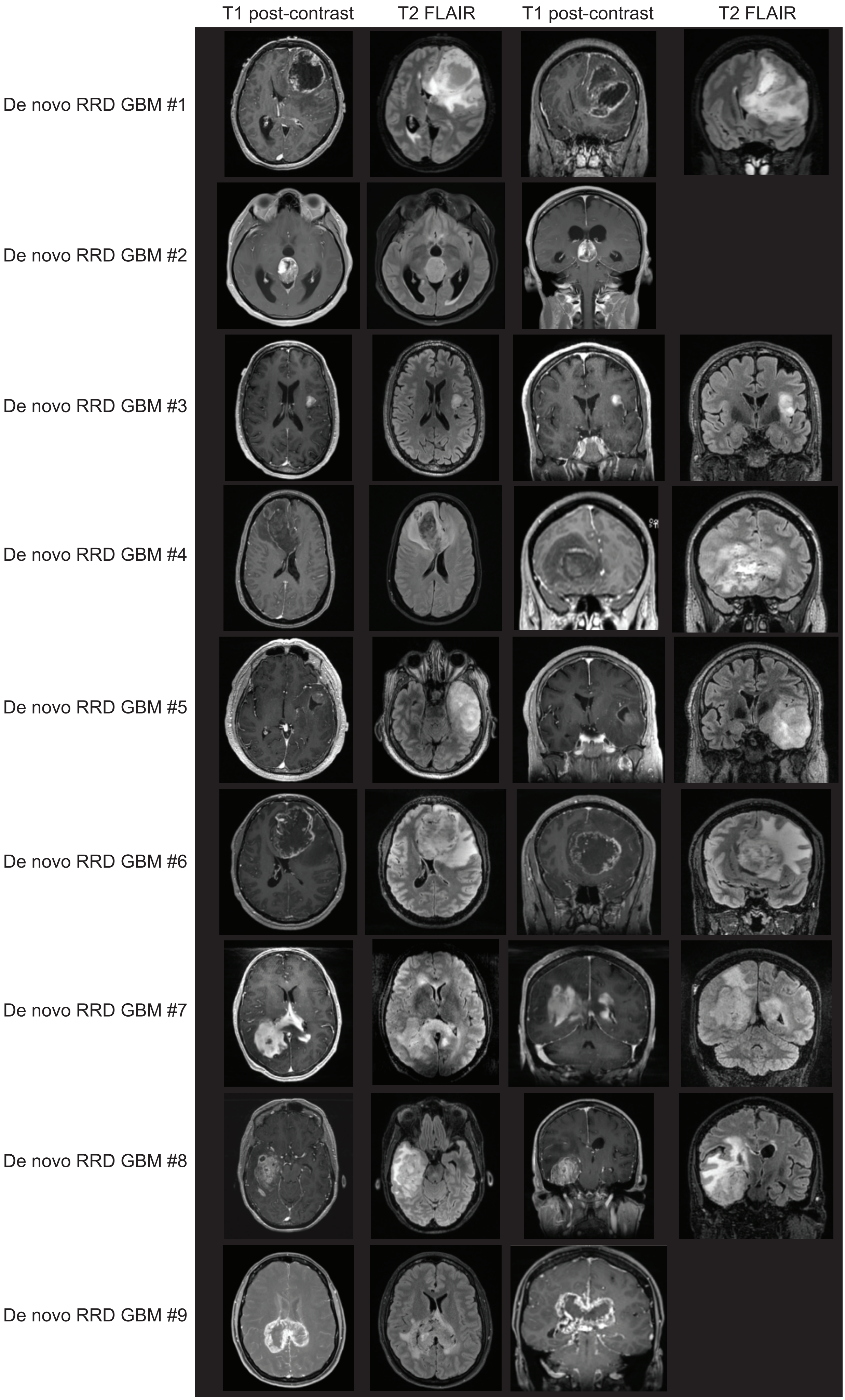

Supplementary Figure 1. Pre-operative imaging features of "De novo replication repair deficient glioblastoma, IDH-wildtype".

De novo RRD glioblastoma #1: -distal 8q, -distal 15q; no focal amplifications or deep deletions

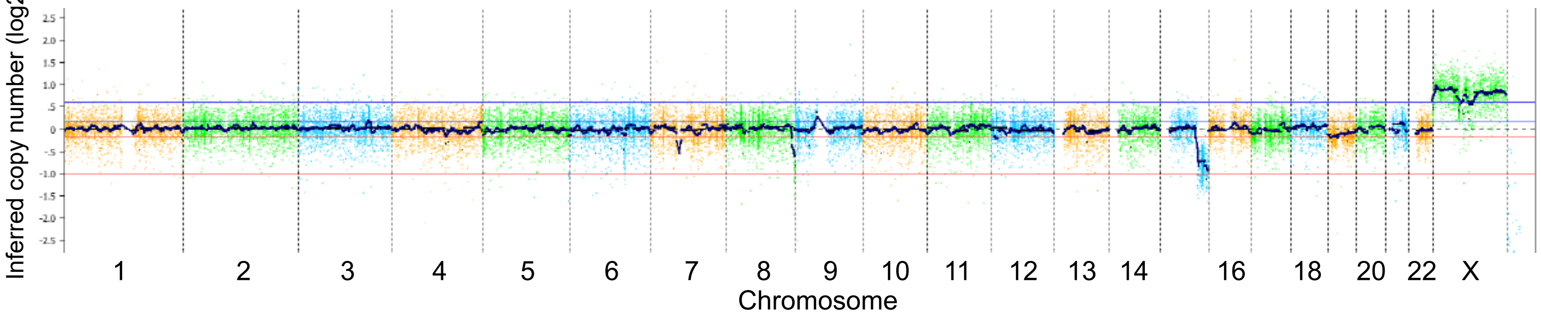

De novo RRD glioblastoma #5: -interstitial 5q, -distal 6p, +proximal 6p, -distal 9p, -distal 13q, -14q, +distal 17q; no focal amplifications or deep deletions

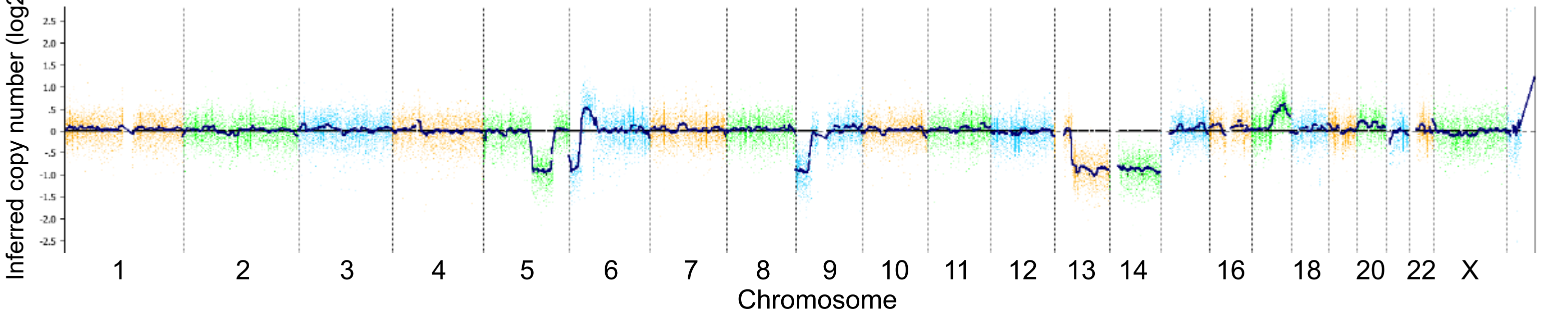

Conventional glioblastoma, IDH-wildtype: +7, -distal 9p, -10, -distal 18q, +19, focal *EGFR* amplification, focal *CDKN2A/B* homozygous deletion

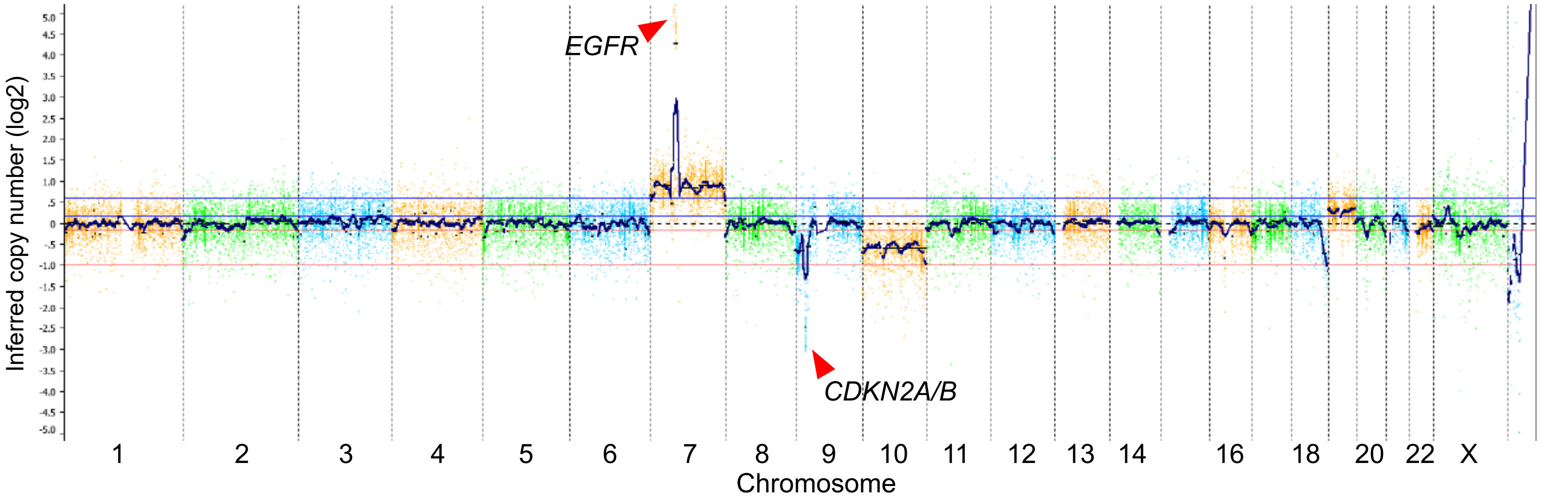

Supplementary Figure 2. Chromosomal copy number plots for two “De novo replication repair deficient glioblastoma, IDH-wildtype” demonstrating a small number of chromosomal gains and losses per tumor, but an absence of focal amplifications and deep deletions and an absence of combined trisomy 7 plus monosomy 10. A prototypical chromosomal copy number plot for a conventional glioblastoma, IDH-wildtype is shown for comparison that has focal high-level *EGFR* amplifcation, focal *CDKN2A/B* homozygous deletion, and combined trisomy 7 plus monosomy 10.

Conventional glioblastoma, IDH-wildtype, with heterozygous *MSH6* frameshift mutation affecting one of two alleles in tumor cells and intact MSH6 protein expression by immunohistochemistry, microsatellite stable (instability at <2% of the 86 evaluated microsatellites), low somatic mutation burden (<5 somatic mutations per Mb), absence of giant cell morphologic features, chromosomal copy number profile = trisomy chromosome 7 + monosomy chromosome 10, genetic alterations = focal *EGFR* amplification, focal *CDKN2A/B* homozygous deletion, *TERT* promoter c.-124C>T mutation, DNA methylation profile = Glioblastoma, IDH-wildtype, mesenchymal type with calibrated score of 0.876 by version 12.5 of DKFZ Molecular Neuropathology classifier

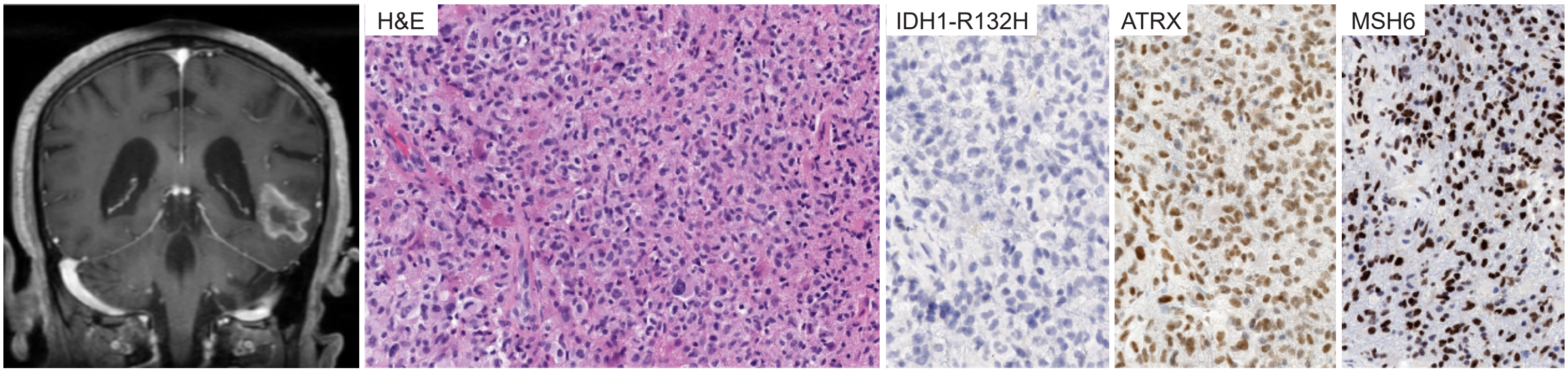

PATHOGENIC AND LIKELY PATHOGENIC ALTERATIONS

VARIANT	TRANSCRIPT ID	CLASSIFICATION	READS	MUTANT ALLELE FREQUENCY
CDKN2A, CDKN2B homozygous deletion	all	Pathogenic	N/A	N/A
EGFR p.S229C	NM_005228.3	Pathogenic	1588	88%
EGFR high level amplification	NM_005228	Pathogenic	~10,000 (>15x)	N/A
MSH6 p.Y397fs	NM_000179.2	Pathogenic	1344	48%
PTEN p.N48fs	NM_000314.4	Pathogenic	368	86%
STAG2 p.R259*	NM_001042749.1	Pathogenic	278	95%
TERT c.-124C>T	NM_198253.2	Pathogenic	1311	52%
TP53 p.C242R	NM_000546.5	Pathogenic	586	46%
Trisomy 7, Monosomy 10	N/A	Pathogenic	N/A	N/A

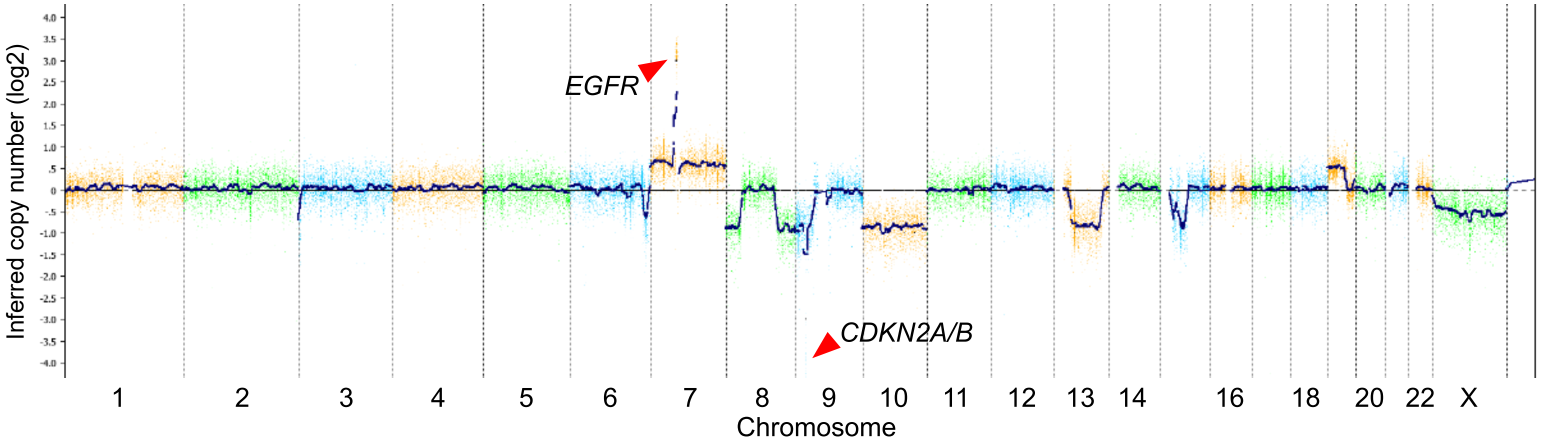

Supplementary Figure 3. Rare primary treatment-naive conventional IDH-wildtype glioblastomas in the cerebral hemispheres of adults contain heterozygous inactivating mutations in a canonical mismatch repair gene affecting one of two alleles and have intact expression of the affected mismatch repair protein in tumor cells by immunohistochemsitry, along with absence of microsatellite instability and absence of somatic hypermutation. Two of the 450 conventional primary treatment-naive IDH-wildtype glioblastomas in this patient cohort (<1%) contained heterozygous *MSH6* frameshift mutations affecting one of two alleles in tumor cells. Both demonstrated typical glioblastoma histology with absence of giant cell morphology and had intact MSH6 protein expression by immunohistochemistry, were microsatellite stable, had low somatic mutation burden, had the combination of trisomy chromosome 7 + monosomy chromosome 10, contained genetic alterations typical of conventional IDH-wildtype glioblastoma (*TERT* promoter mutation, *CDKN2A/B* homozygous deletion, *EGFR* amplification), and had DNA methylation profiles that aligned with reference methylation classes of adult-type glioblastoma, IDH-wildtype with high calibrated scores.
